# The Impact of FGF Expression in Breast Cancer

**DOI:** 10.3390/cimb48101051

**Published:** 2026-10-12

**Authors:** Robert Wieder

**Affiliations:** 1 Rutgers New Jersey Medical School, Newark, NJ 07103, USA; wiederro@njms.rutgers.edu; 2 The Cancer Institute of New Jersey, Newark, NJ 07103, USA

**Keywords:** breast cancer progression, fibroblast growth factor, prognosis, intracellular expression

## Abstract

Fibroblast growth factors (FGFs) are ubiquitous growth factors, with functions in all organs and cells of the embryo and the post-partum mammal, and roles in all aspects of normal and pathologic physiology. They belong to a large family of proteins categorized into seven subfamilies with 23 members. FGF molecules initiate signaling through varied and interacting mechanisms that result in a highly diverse spectrum of multifunctional effects. FGF molecules induce their effects by binding to transmembrane tyrosine kinase receptors to initiate canonical signal transduction pathways, and by internalization of the FGF/FGF receptor (FGFR) complexes and directing intracellular non-canonical signaling in the cytoplasm and the nucleus. FGF proteins expressed by a cell can also engage in non-canonical intracellular signaling in the cytoplasm, in the nucleus, or by undergoing extracellular export and initiating autocrine, receptor-mediated signaling. In the mammalian breast, FGF family members play significant roles in embryonic and postnatal mammary gland development and the normal female mammalian life cycle. In breast cancer (BC), the intracellular detection of FGF family members has been associated with both cancer-promoting and cancer-inhibitory effects. The goal of this review is to deconvolute the often contradictory published data resulting in these differing impacts of FGF expression on BC. Within the context of these variables, there is a potential for an extraordinarily high number of combinatorial interstudy scenarios, interactive variables, experimental designs, and circumstance-specific responses to potential signal-initiating scenarios, rendering generalizations difficult. However, the available evidence suggests that pro-malignant FGF effects in BC are generally mediated by the combined effects of intracellularly initiated signaling and cell membrane FGFR-mediated autocrine or paracrine canonical signaling, together with crosstalk with other external factors. In contrast, the anti-cancer effects of intracellular FGF molecules are primarily mediated by intracellular signaling that results in the redifferentiation of cancer cells and adhesion-mediated crosstalk with the stroma.

## 1. Background

Members of the fibroblast growth factor (FGF) family play essential functions in mammary gland development and in normal and malignant physiology. In breast cancer (BC), the expression of FGF family members has been associated with both less and more aggressive malignant behavior. This duality is not well understood. This review outlines differences in key elements of published studies with contrasting conclusions on the effects of FGF expression, and reconciles the likely differences in molecular processes and experimental designs and measurements that generate the dichotomy.

The ubiquitous and exceptionally heterogeneous functions of FGF proteins are regulated at multiple levels along the spectrum of molecular and cellular processes. The FGF family comprises 23 members allocated to 3 major categories, canonical, endocrine, and intracellular [1] (Table 1). Within these major categories, FGFs fall into seven subfamilies. The canonical category encompasses five FGF subfamilies: subfamily 1, comprising FGF-1 and -2; subfamily 4, comprising FGF-4, -5 and -6; subfamily 7, comprising FGF-3, -7, -10 and -22; subfamily 8, comprising FGF-8, -17 and -18; and subfamily 9, comprising FGF-9, -16 and -20 [1,2,3]. The endocrine FGF category, with low affinity for FGF receptors (FGFRs), consists of the FGF subfamily 15/19, comprising FGF-15/19 (FGF-19 is the human ortholog of mouse FGF-15), and FGF-21 and -23 [4]. The intracellular category, comprising the FGF-11 subfamily, also known as the FGF Homology Factors (FHF), contains FGF-11, -12, -13 and -14 [1,2,3]. The FGF-11 family members are non-FGFR signaling proteins that were initially investigated as cofactors for voltage-gated sodium channels, among other roles [1,2,3]. FGF family members with reported roles in the spectrum of mammary gland development, normal physiologic and malignant function include FGF-1, -2, -3, -4, -5, -7, -8, -9, -10, -11, -13, -17, -18, -19, -21, -22 and -23 [2,3,5,6,7,8,9,10,11,12,13,14,15] (Table 1).

Individual FGFs are regulated by heterogeneous transcriptional, splicing, and translational processes, differential and varied expression in different organs, cell types, and stages of differentiation [4,16,17], and differential extracellular export and intracellular localization [18,19,20,21,22]. FGF proteins initiate extracellular signaling through four membrane receptors differentially expressed and spliced [2,23,24,25], and often mutated or amplified.

The four receptor classes have differential affinities to different FGF subfamily members and their preferential binding cofactors, heparan sulfate proteoglycans (HSPGs) and Klotho [3,26,27,28,29]. FGFs activate receptor-mediated signaling through four major molecular pathways [1,2,30,31], endowing different signaling and transcription programs [32,33]. They are also regulated through epigenetic differences [33], isoform differences [18], mutation-induced receptor differences [29,34,35] and interactions with other signaling pathways [36,37].

Receptor-mediated signaling by specific FGF family members plays roles in normal and malignant breast physiology, covered elsewhere [3,29,35,38,39,40,41,42,43,44]. Once formed, FGF/FGFR complexes are internalized into the cytoplasm and the nucleus, where they undertake additional signaling [45,46,47]. These processes and their varied effects have been delineated in comprehensive reviews [2,3,4,48,49,50,51,52].

FGF proteins expressed by cells also initiate varied modes of signaling [18,19,21,22]. They undergo export, where they initiate receptor-mediated autocrine, paracrine or endocrine receptor-mediated signaling, as above [22,53]. They also initiate intracellular signaling through less well-defined mechanisms [19,20,45,54,55], which include crosstalk with nuclear steroid receptors, adding an additional level of complexity to their signaling [46,47].

Both FGF expression and receptor-mediated signaling are important in mammary stem cell function [56,57,58], duct formation, cell migration, morphogenesis, and differentiation [58,59,60,61,62,63,64], and dynamic growth and involution of breast ducts in the mammalian female life cycle [62,65,66,67,68]. FGF signaling is also important in malignancy [69], cancer stem cell survival, maintenance and function [70,71,72,73,74,75]. Signaling is mediated by both autocrine effects, initiated by receptor binding of exported FGF isoforms or by FGF proteins generated by sources in the microenvironment [76], and by intracellularly mediated non-canonical effects, through less understood mechanisms [18,19,20,45,54,55].

The expression of FGF proteins in BC has been associated with promotion of the malignant phenotype in preclinical and clinical settings [77,78,79,80]. Conversely, the expression of FGF has also been shown to induce a less malignant phenotype in BC cells in preclinical investigations, to exhibit a gradual loss with malignant progression in patient tumors, and to be associated with a better prognosis in patients [81,82,83,84].

The context of the exceptionally large number of variables generated by the potential interactions of canonical and noncanonical signaling by FGF family members in BC cells and the tumor microenvironment (TME) is key to determining the behavior of the cancer cell. To begin to understand the often opposing and context-dependent effects of the expression of FGF family members in BC, it is necessary to first present a historical overview of FGF signaling as well as more recent updates that provide background, context and insight into the frequently opposite reported effects of FGF family expression in BC. In the first part of the BC effects section, the manuscript reviews the data supporting the pro-malignant effects of FGF family members expressed in cancer cells. It delineates the data on how these pro-malignant effects are mediated by the intracellular signaling of newly expressed FGF molecules combined with FGFR-mediated canonical signaling initiated by autocrine binding of exported FGF and microenvironmentally derived paracrine FGF molecules, the subsequent internalization of the FGF/FGFR complexes, and their intracellular, non-canonical signaling. Secondly, the manuscript reviews the data supporting the anticancer effects of intracellularly expressed FGF molecules that inhibit and redifferentiate BC cells and promote antimalignant effects when combined with adhesion-mediated integrin receptor signaling.

## 2. FGF Receptor-Mediated Signaling

### 2.1. Classical Receptor-Initiated FGF Signaling

Classical FGF signaling is mediated through 4 transmembrane tyrosine kinase receptors, FGFR1-4 [3]. FGF-family members bind these receptors with the assistance of cofactors that include HSPGs for canonical FGF-subfamilies and Klotho for FGF-subfamilies 15/19 [3,85,86,87]. FGFR1-3 have alternate splice variants that result in epithelial signaling for the FGFR1-3IIIb (FGFR1b-FGFR3b) isoforms, and mesenchymal signaling through the FGFR1-3c isoforms [25,88,89,90,91], affecting cancer cell behavior during the multiple steps of progression [92]. Various FGFR isoforms and polymorphisms have been associated with varying BC aggressiveness [93,94,95,96,97,98].

FGF receptors heterodimerize, transphosphorylate their intracellular C-terminal tyrosines, and initiate intracellular signaling through four major pathways: the RAS-Mitogen-Activated Protein Kinase (MAPK), Phosphatidylinositol-4,5-Bisphosphate 3-Kinase (PI3K)-AKT, Phospholipase C*γ* (PLC*γ*), and Signal Transducers and Activators of Transcription (STAT) signaling pathways [1,2,3,30,31]. Receptor-mediated signaling induces multiple, often context-specific, and sometimes opposing physiologic functions, including proliferation vs. cell cycle arrest, motility activation vs. inhibition, invasion promotion vs. invasion inhibition, cell survival vs. cell death activation, differentiation, branching elongation and morphogenesis, resistance to toxins, cancer initiation and progression vs. cancer cell dormancy, among many others.

FGFR-mediated signaling in MCF-7 cells by FGF-2 from the canonical FGF-1 subfamily, FGF-3 and FGF-10 from the canonical FGF-7 subfamily, FGF-4 from the canonical FGF-4 subfamily and FGF-19 from the endocrine FGF 15/19 subfamily results in unique biological responses [99]. FGF-2 and FGF-4 have the broadest kinome responses, FGF-10 generates a moderate response, and FGF-3 and FGF-19 have modest responses. Complex combinations of responses to different FGF molecules are influenced by heparin, do not correlate with FGFR levels, and result in different biological responses in different cell lines [99]. Activation of the Extracellular Signal-Regulated Kinase (ERK) pathway alone does not necessarily lead to proliferation, and activation of the PI3K pathway does not necessarily lead to the commonly associated epithelial–mesenchymal transition (EMT) [99]. Different FGF family members affect cell proliferation and EMT differently and unpredictably in different cell types, underscoring the variable correlations between pathway and kinome activation [99].

**Figure 1:**
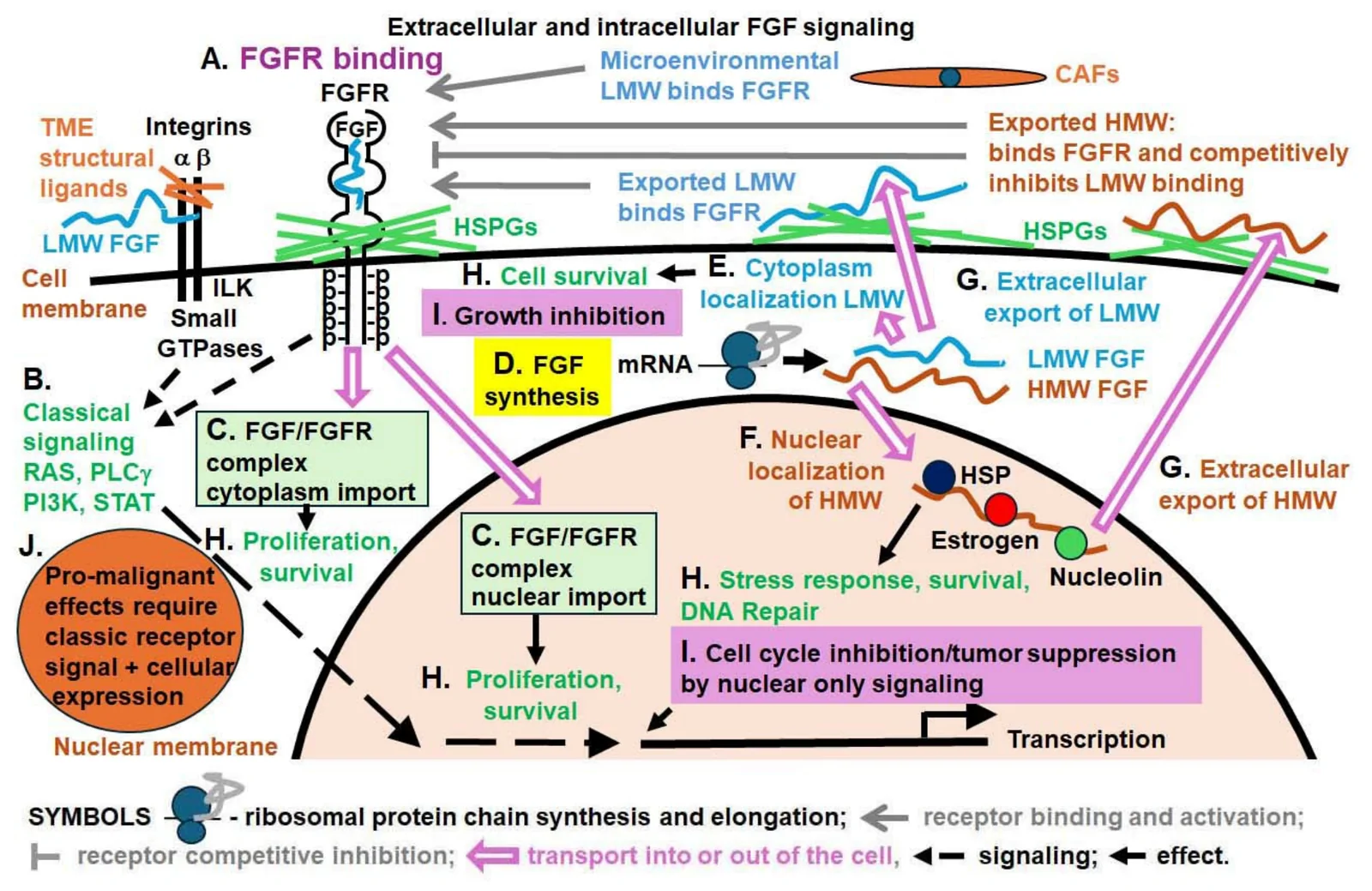
Graphic summary of extracellular and intracellular FGF family localization and signaling. (A) Exported and paracrine FGFs bind FGFRs and integrins. (B) Receptor binding initiates classical signal transduction. (C) FGF/FGFR complexes are imported into the cytoplasm and the nucleus. (D) Protein synthesis of LMW and HMW FGF translation variants. (E) Cytoplasmic localization of LMW FGF. (F) Nuclear import of HMW FGF. (G) LMW and HMW FGF are exported extracellularly and deposited on HSPGs. (H) Activation of stress response, survival and proliferative signaling; (I) Growth suppression by cytoplasmic and nuclear endogenously synthesized FGFs; (J) Malignant transformation requires a combination of FGFR-initiated classical signaling and intracellular FGF expression. Abbreviations: CAFs—cancer-associated fibroblasts; TME—tumor microenvironment; TME ligands (e.g., fibronectin, collagen, laminin); ILK—integrin-linked kinase; FGF—fibroblast growth factor; LMW—low molecular weight FGF; HMW—high molecular weight FGF; FGFR—fibroblast growth factor receptor; HSPGs—heparan sulfate proteoglycans; small GTPases—small guanosine triphosphatases (e.g., Rho, Rac, cdc42); RAS—rat sarcoma small GTPase; PLCγ—phospholipase C-gamma; PI3K—Phosphatidylinositol-3-Kinase; STAT—Signal Transducers and Activators of Transcription; HSP—heat shock protein.

FGF-2 both increases and decreases cell proliferation, supporting the evidence that FGF signaling interacts with additional growth factors and structural proteins co-signaling in the execution of their biologic effects [39,40,99,100]. In fact, FGF-1, FGF-2 and FGF-9 require direct binding to the classical ligand-binding site of integrin αvβ3 for their receptor-mediated signaling and cancer-promoting effects through activation of Fibroblast Growth Factor Receptor Substrate 2 (FRS2)α and ERK1/2, DNA synthesis, migration, and invasion [101]. In contrast, FGF-2-initiated signaling combined with microenvironmental fibronectin-initiated signaling through integrin α5β1 induces cell cycle inhibition and dormancy in BC cells [39,40]. The volume of data and published studies and reviews on the complexity of FGF family signaling is exceptionally large, with the references included here representing just a few of the historically relevant research articles and reviews on signaling by FGF family of proteins, their effects in cancer, and, specifically, in BC [18,29,39,50,102,103,104,105,106,107,108,109,110,111,112]. Figure 1 depicts a simplified schematic of canonical and non-canonical FGF signaling outlined in Section 2 and Section 3.

### 2.2. Receptor-FGF Complex Internalization and Direct Signaling

Canonical, classic, receptor-mediated signaling is only one part of FGF family-mediated signaling. Once FGF family members are bound to FGFRs, the FGF/FGFR complexes are internalized into the cytoplasm and the nucleus under conditions of stress through endocytosis [20] to promote cell survival and engage in intracellular signaling [113,114,115,116,117,118]. Endocytosis into the cytoplasm can be both dependent and independent of clathrin-coated pits, and may also involve caveolin [119,120]. Vesicle-mediated internalization requires an electrical potential involving a proton pump and a Na^+^/K^+^-ATPase, PI3 kinase activity and Heat Shock Protein-90 (Hsp90), and is most efficient in the G_1_ phase of the cell cycle [120,121,122,123]. FGF binding activates the FGFR and stimulates clathrin-coated pits (CCPs) to engage in endocytosis through activation of Src and its target, the scaffolding protein epidermal growth factor receptor pathway substrate 8 (Eps8) [124]. Src and Eps8 are necessary for clathrin-mediated endocytosis of FGFR and downstream ERK signaling, early endosome uptake and degradative recycling [124]. Internalization of FGF-1 and FGF-2 into the cytoplasm by endocytic vesicles, and into the nucleus through nuclear pores [125], is strictly dependent on the intracellular binding partner Leucine-Rich Repeat-Containing Protein (LRRC)59 with an endoplasmic reticulum and a nuclear envelope localization sequence, Ran GTPase, karyopherin-α1 (Kpnα1), Kpnβ1, and CEP57/Translokin for FGF-1, but is independent of LRRC59 in the case of FGF-2 [125]. Once internalized, FGF-1 is phosphorylated by Protein Kinase C (PKC) in the cytoplasm and the nucleus, and binds both cytoplasmic and nuclear proteins [117,126]. Once phosphorylated in the nucleus, FGF-1 is exported to the cytoplasm, where it is rapidly dephosphorylated [126].

Internalized FGF/FGFR complexes promote proliferation. FGF-2/ or FGF-1/FGFR1 complexes translocate to the cytosol, from where they are transported to the nucleus through importin β1-mediated mechanisms [115,127] and Ras-related protein Rab-5 (Rab5) [128], where they induce c-Jun and play an important role in cell cycle progression [118,127]. FGF/FGFR1 complexes in the nucleus stimulate proliferation in both embryonic and adult neuronal cells by enhancing c-Jun1 expression, rRNA transcription and DNA synthesis, mostly transiently in G_1_ [120]. The nuclear translocation of exogenous 18 kDa FGF-2 has been found to be necessary for a full mitogenic response in target cells [129]. Internalized exogenous FGF-2 interacts with cytosolic translokin, a ubiquitous protein which co-localizes with microtubules and which mediates nuclear transport of cytoplasmic low molecular weight (LMW) FGF-2 [130].

Nuclear FGFR1 signaling combines with classical membrane receptor signaling to induce a combined positive feedback system for the expression of FGF-2 and other target genes, such as tyrosine hydroxylase, by activating sequence-specific transcription factors (ssTFs) and the common coactivator, CREB-Binding Protein (CBP) [131]. FGFR1 co-localizes in the nuclear matrix with splice factors, aids in acetylation of histones at active promoters, and recruits phosphorylated RNA Pol II to globally stimulate mRNA transcription and processing in multi-gene programs at active sites of different chromosomes for cell growth and differentiation [131,132,133]. FGFR1 binds Microtubule-Associated Protein-2 (MAP2), c-jun [127,131], and Ribosomal S6 Kinase (RSK)1, which are responsible for phosphorylating histones and regulating transcriptional activities [134].

Once translocated to the nucleus, LMW FGF-2 complexed with FGFR binds nucleolin, histone H1, ribosomal protein P0, and Upstream Binding Factor (UBF), an essential transcription factor for rRNA transcription, phosphoglycerate kinase 1 and 2 genes, and stimulates mRNA [135] and rRNA transcription in vitro and in vivo [120,136,137,138]. FGF-2 in the nucleus interacts with and stimulates CK2, which leads to phosphorylation of nucleolin [139]. Both high molecular weight (HMW) FGF-2, which translocates to the nucleus after synthesis, and LMW FGF-2, which localizes in the cytoplasm after synthesis, bind to the ribosomal protein L6/TAXREB107 and may be involved in the pre-ribosomal assembly process [136,140].

Nuclear FGFRs may potentiate RSK2 activities during cell-cycle progression [113,120]. FGF/FGFR1 complexes in the nucleus stimulate proliferation in both embryonic and adult neuronal cells by enhancing c-jun1 expression, rRNA transcription and DNA synthesis, mostly transiently in G1 [120]. Nuclear FGF-2/FGFR1 complexes are involved in mRNA processing during neuronal differentiation, by associating with snRNPs during neuronal differentiation [131]. In neurons, nuclear FGFR1 affects the expression of Neurofilament-L, neuron-specific enolase MAP2, c-jun and cyclin D1 [127,131]. Nuclear LMW FGF-2 associates with RSK2 in a cell cycle-dependent manner [113], and when complexed with FGFR1, is involved in mRNA processing during neuronal differentiation by associating with snRNPs [131].

In addition to promoting cell cycle progression, internalized FGF/FGR complexes also inhibit cell death. FGF-1 and FGF-2 internalized by receptor-linked endocytosis have antiapoptotic activity that does not depend on FGFR activation or signaling. The FGF-1 anti-apoptotic effects depend on its phosphorylation and binding to nucleolin [20]. FGF-2 is not phosphorylated by PKC∆ and is present inside the nucleus for longer durations than observed for FGF-1, which is rapidly exported after phosphorylation [20]. The anti-apoptotic effects of FGF-2 are thus higher than those of FGF-1, commensurate with its longer duration of stay inside the nucleus [20].

FGF molecules bound to cell surface HSPGs can also be endocytosed and allocated to endosomes, and later lysosomes [141,142], but internalized FGF remains long-lived [118]. The cell’s microenvironment initiates nuclear events by activating FGFR-mediated signaling through contact with stored FGFs on cell surfaces, paracrine FGF from other cells, hormones and second messengers [131]. The roles of FGF internalization and the intracellular activity of some FGFs are discussed comprehensively in the context of the known signaling induced by FGF [118,136].

The next section outlines evidence of intracellular signaling by FGF isoforms expressed inside cells and translocated to the nucleus [143,144]. FGF isoforms synthesized in the cell that localize in the cytoplasm, however, need to be exported first and then reinternalized when complexed with receptors in order to translocate to the nucleus [120]. However, nuclear translocation from the cytoplasm may not be necessary for FGF survival effects, since data have demonstrated antiapoptotic effects of an FGF-1 species with disrupted nuclear localization sequences (NLSs) after it was presented exogenously and internalized to the cytoplasm by cells exposed to pro-apoptotic pressure [145].

### 2.3. Key Takeaways from Section 2

1. FGF family members, FGFRs, with assistance from low-affinity cofactors HSPGs or Klotho, initiate classical signaling, primarily through four classical pathways.
2. Canonical FGF/FGFR and structural protein/integrin signaling cooperate to induce varied effects on proliferation, motility, survival and dormancy.
3. FGF/FGFR and FGF/HSPG complexes are internalized to the cytoplasm and nucleus under cell stress, and initiate non-canonical proliferative and survival signaling.
4. The exceptional heterogeneity of FGFR-mediated signaling by members of the FGF family is derived from a very high number of potential scenarios from the combined interactions of variables from each component of the system, outlined in Table 2.
5. Only a fraction of the potential scenarios resulting from the interaction has been investigated. A number of comprehensive reviews over the years have addressed the details [2,30,118,120,136,144,146]. The following sections review the classic original studies that provided much of our understanding of FGF family signaling.

## 3. FGF Expression and Intracellular Signaling

### 3.1. Intracellular Trafficking

In addition to the transport of FGF/FGFR cell membrane complexes to the cytoplasm and nucleus, some isoforms of FGF-1, FGF-2, FGF-11, FGF-12, FGF-13 and FGF-14, which are synthesized in the cytoplasm, also lack signal peptides, but are capable of being imported into the nucleus through NLS [136,143,146,147,148,149,150,151,152,153,154,155,156]. FGF-3 also has NLS that permits translocation to the nucleus, but also has a signal sequence that enables extracellular export [153]. Some members of the FGF family in other than human species, including FGF-1, FGF-5, FGF-8, and FGF-11 to FGF-14, have alternate splice forms that permit nuclear localization [136]. FGF-11–FGF-14, the FGF Homology Factors, share less than 30% amino acid sequence homology with other FGFs, do not stimulate FGFRs but do signal intracellularly [118,156].

**Figure 2:**
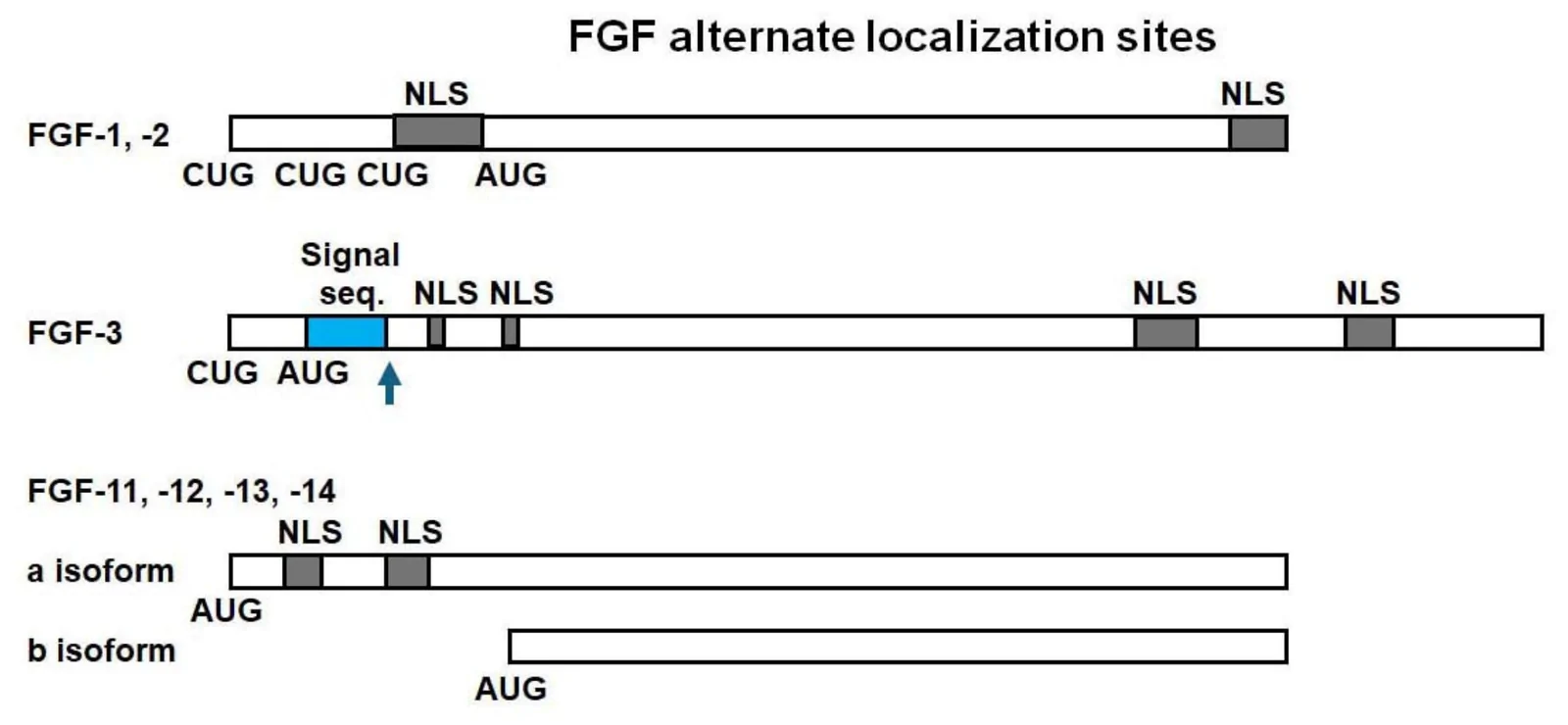
Subcellular localization sequences in FGF-1, -2, -3, -11, -12, -13 and -14 mRNAs. FGF-1 and FGF-2 are predominantly translation-initiated at the AUG codon, giving rise to approximately 18 kD cytoplasm-localizing proteins [LMW species]. Three CUG-initiated protein entities of 22, 22.5 and 24 kD [HMW species] localize in the nucleus [120,136,146,148,153,154,155,156]. A 34 kD CUG-initiated species has also been reported [154]. The C-terminal NLS of FGF-1, -2 and -3 are also required for efficient nuclear localization of HMW species [152,153,158,160,162]. FGF-3 translation is primarily initiated at the CUG codon. A signal sequence at the N-terminal AUG-initiated protein supports extracellular secretion, and competition between the signal peptide and the bipartite NLS determines the fate of FGF-3 extracellular vs. nuclear transport [153,158]. Cleavage of the signal seq. of the full sequence, an approximately 31 kD protein, results in nuclear translocation of the cleaved, approximately 21 kD protein. FGF-11–FGF-14 have two isoforms, a and b, varying in reported MW from 19 to 25 kD, generated by alternate usage of two different first exons, but only the a isoform localizes to the nucleus, requiring both NLSs for efficient translocation [156,167,168]. Abbreviations: NLSs—nuclear localization sequences; Signal seq.—Classical Golgi apparatus-mediated extracellular export; **↑**—cleavage site of Signal seq.; CUG—cytosine–uracil–guanine translation-initiation codons; AUG—adenine–uracil–guanine initiation codons.

FGF-1 and FGF-2 LMW isoforms are initiated by the classical AUG translational initiation sequences and the HMW isoforms are initiated by upstream CUG alternate translation initiation sites [20,120,146,148,153,154,155,156], primarily in stressed non-transformed and cancer cells [136]. The expression of the HMW isoforms is activated at the translational level in transformed and heat-stressed fibroblasts, while unstressed non-transformed cells express almost exclusively the AUG-initiated 18 kDa isoform, supporting the potential role for HMW forms as survival factors [157] (Figure 2).

FGF-3 expressed in prenatal mice has a predominantly CUG-initiated translation site that allows it to translocate to the nucleus and, specifically, the nucleolus, in addition to being exported extracellularly [153,158] (Figure 2). It was also reported to have a role in carcinogenesis in a mouse model [69]. Competition between the effects of the signal sequence and NLS determines whether the FGF-3 protein molecule is exported to the outside of the cell or translocated to the nucleus [153]. The NLS segments contain Glu/Arg repeats with mono- and di-methylated Arg residues [159] upstream of the classical AUG translation initiation site, with the methylation on some arginine residues being necessary for HMW FGF-2 nuclear translocation [160] and stability [161]. The C-terminal domains of isoforms of FGF-1, FGF-2 and FGF-3 contain a bipartite NLS as well, and both the N-terminal and C-terminal NLSs are required for efficient nuclear localization [152,153,158,160,162]. The LMW FGF-1 and FGF-2 species that do not have nuclear localization sequences have to be exported and bind FGF receptors in order to be internalized to the nucleus [120].

The HMW FGF isoforms are not exported efficiently to the extracellular space, but are mostly imported into the nucleus, where they have a variety of functions in various systems [136,146,148,153,154]. However, estrogen and 27 kDa human HSP induce HMW FGF-2 secretion, and secreted HMW FGF-2 was reported as mitogenic to breast adenocarcinoma cells through external signaling in one study [163]. Indeed, recombinant HMW FGF-2 binds FGFR1 with high affinity, which can be competitively inhibited by LMW FGF-2 [164]. HMW FGF-2 isoforms have been reported to have both stimulatory and inhibitory effects on cells. They can activate the canonical FGFR/MAPK pathway and induce mitogenic activity, similar to the 18 kDa FGF-2 isoform, but less effectively [165]. In contrast, receptor-mediated HMW FGF-2 inhibits the migration of endothelial and estrogen receptor (ER)+ MCF-7 BC cells [163], an effect mediated by the ER [166].

FGF-11, FGF-12, FGF-13 and FGF-14 have two differentially spliced RNA first exons that serve as templates [156,167]. The HMW form contains a basic amino acid-rich sequence that resembles an NLS and is able to accumulate in the nucleus in an NLS-dependent manner [reviewed in 118, 120] (Figure 2). These proteins have primary neuronal roles in cells and bind to intracellular domains of voltage-gated sodium channels (VGSCs) and to a neuronal MAP kinase scaffold protein, islet-brain-2 (IB2), and have mutations that account for several neurological abnormalities [156,167,168]. However, roles for FGF-11 and FGF-13 in promoting cancer progression have been reported and are discussed below [169,170,171].

Interactions between FGF-2, localized to the nucleus through a cryptic NLS, and Apoptosis-Inhibitor 5 (API5), upregulated in certain cancers and correlated with poor prognosis, are important for mRNA export from the nucleus, especially mRNA containing eIF4E sensitivity elements such as c-Myc and cyclin D1 [172]. A cDNA microarray study in NIH 3T3 fibroblasts, expressing the different FGF-2 forms HMW FGF-2 and LMW FGF-2, induced the expression of different sets of genes and suggested that HMW FGF-2 forms may function as inducers of growth inhibition and tumor suppression [173,174].

Once in the nucleus, FGF-1 can be serine phosphorylated by PKC∆ and rapidly exported to the cytosol by Exportin-1 and its co-factor Ran-GTP through a C-terminal Leu-rich nuclear export sequence (NES) in a leptomycin B-sensitive pathway [175], where it is degraded [126,175,176]. In the nucleus, the phosphorylation and export of FGF-1 requires binding of nucleolin through its heparin-binding site [176], binding and activation of Casein Kinase (CK)2, resulting in phosphorylation of nucleolin in cycling cells, but not in quiescent cells, where CK2 is not detectable in the nucleus and nucleolin is dephosphorylated [139].

### 3.2. Intracellular Signaling

The following data is a partial summary of what is known about intracellular signaling by FGF species. Intracellular FGF family member-generated signaling must be considered as part of a complex set of interacting events that take place in both the cytoplasm and in the nucleus in conjunction with extracellular signaling. The different FGF species synthesized in the cytoplasm either localize primarily in the cytoplasm (LMW) or localize in the nucleus (HMW), and are capable of being exported extracellularly (mostly LMW but also observed for HMW species), as described above. Cytoplasmic and nuclear internalization of LMW FGF complexed with FGFRs are also discussed above.

In the cytoplasm, various FGF family members have been found to interact with a number of proteins. These include FGF-1 intracellular binding protein, mortalin/GRP75 of the hsp70 family, the serine/threonine kinase CK2, ribosome binding protein p34, or FGF-2 binding cytoplasmic translokin localized to microtubules [118]. The LMW FGF-2 interaction with CK2 stimulates it to phosphorylate nucleolin [139]. Of course, the effects of LMW FGF-2 are not only cytoplasmic but are also mediated by its extracellular export, FGFR binding and classical signal transduction, followed by internalization and FGFR intracytoplasmic direct signaling by target binding.

Intranuclear signaling is mediated by HMW FGFs synthesized by the cell as well as the LMW FGF-FGFR internalization to the nucleus, where both species bind to and activate ligands and initiate direct signaling. The following results of some of the many investigations describe direct signaling by nuclear FGFs and their receptors.

Enforced intracellular expression of HMW or LMW FGF-2 in NIH3T3 cells demonstrated different gene expression patterns in the two transfectants and suggested that HMW FGF-2 induced growth inhibition and tumor suppression activities [174]. Genes preferentially upregulated in HMW FGF cells included select ones from the categories of growth inhibition and tumor suppression, cell adhesion/extracellular matrix (ECM) molecules, development/differentiation, DNA binding, RNA binding and processing, biosynthesis and binding proteins, mitosis, protein binding, structural proteins, translation, transport/energy and others with unknown functions. Genes preferentially downregulated in HMW FGF-2 expressing 3T3 cells were from the categories classified as cell proliferation/mitogenesis, angiogenesis, RNA binding/biogenesis, DNA binding, biosynthesis, metabolism, binding protein, cellular component and unknown functions [174].

Nuclear FGF-3 is involved in ribosomal biogenesis [120]. It interacts with nucleolar FGF-3 binding protein (NoBP, or Ebp2p) in pre-rRNA processing and is essential for the synthesis of the 60S ribosomal subunit and ribosomal protein S2 (rpS2), a component of the small subunit of the ribosome [177,178]. It binds nucleolin and the growth and differentiation factors midkine and pleiotropin, which are upregulated during the late G_1_/early S phase. However, an FGF-3 species engineered to eliminate its extracellular export sequences, forcing it to only localize to the nucleus and nucleolus, inhibited DNA synthesis and cell proliferation [179] by blocking NoBP and rpS2 activity, blocking ribosomal biogenesis and cell proliferation [177]. NoBP overexpression confers a proliferative effect on NIH 3T3 cells and can counteract the inhibitory effects of nuclear FGF-3, supporting the role of NoBP in controlling proliferation in cells [177].

In addition to promoting cell cycle progression, intracellular FGFs can act as survival factors. Preferential expression of HMW FGF-2 has been reported in malignant and stressed cells [162,180]. HMW nuclear forms of FGF-2 physically interact with the anti-apoptotic putative transcription factor FGF-2-Interactive Factor [FIF]2 and may be important in a cell’s response to stress [118,181]. Nuclear HMW FGF-2 modulates IL-6 and tyrosine hydroxylase transcription [131,182] and interacts with the anti-apoptotic putative transcription factor FGF-2 Interacting Factor [FIF]2, potentially indicating a response to stress [120,181]. HMW FGF-2 also associates with small nuclear ribonucleoprotein particles (snRNPs) and Survival of Motoneuron (SMN) in the nucleus [183]. SMN is a protein that functions as an assembly and splicing machine and is found in both the cytoplasm and the nucleus [183]. One of the subunits of the splicing factor 3a was identified as a binding partner for HMW FGF-2 [184].

These events all suggest pro-survival and pro-proliferative effects. Indeed, human astrocytic tumors express high levels of HMW nuclear FGF-2, which correlates with poor prognosis [185]. Enforced expression of HMW FGF-2 in HeLa cells upregulates the DNA-repair DNA-Dependent Protein Kinase (DNA-PK) enzyme subunit, inducing radioresistance, and is associated with radioresistance in patients with cancer [120,186]. However, when FGF-1 nuclear translocation is incapacitated by mutation or deletion of its NLS, or knockdown of its nuclear transport protein Leucine-Rich Repeat-Containing Protein (LRRC)59, transfected mutated FGF-1 is still able to act, as its wild-type form, to increase cell viability, mitochondrial membrane potential and reduce caspase 3/7 activity and Poly(ADP-Ribose) Polymerase PARP cleavage in cells stressed to undergo apoptosis [145].

Enforced combined expression of HMW and LMW FGF-2 induces immortalization of normal cells in vitro [154,187,188]. Acting together with FGFR1 in the nucleus, FGF-2 is a survival and proliferative factor in malignancy [131,189]. With respect to FGF-1, while a species expressing an NLS is able to translocate to the nucleus and trigger DNA synthesis in cells not expressing FGFRs, the mitogenic effects of FGF-1 are only achieved in cells expressing FGFR [146,147,190]. However, in contrast to the effects observed with FGF-1 and FGF-2, an engineered, non-secreted, intracellularly expressed, nuclearly localized FGF-3 inhibits DNA synthesis and proliferation [179].

LMW forms of FGF-1 can induce NIH 3T3 transformation as measured by enhanced saturation density, growth in soft agar, and growth in low serum [173]. Both low level expression (0.41 ± 0.01 ng/10^6^ cells) and high level expression (1.73 ± 0.09–3.0 ± 0.09 ng/10^6^ cells) of a combination of LMW and HMW FGF-2 in NIH-3T3 cells through retroviral transduction induces higher rates of proliferation, higher rates of Bcl-2 expression, prevention of serum starvation- and cytotoxicity-induced apoptosis, and diminished 2D clonogenicity than observed in control vector-transduced NIH 3T3 cells (0.21 ± 0.03 ng FGF-2/10^6^ cells) [103]. The induced Bcl-2 expression was not reversed by neutralizing antibody to FGF-2, suggesting an intracellular antiapoptotic effect. Only the high FGF-2 level-expressing cells exported LMW FGF-2 to the extracellular space, formed foci in 2D culture and anchorage-independent colonies in soft agar. The focus formation in 2D culture was reversible by neutralizing antibody to FGF-2, suggesting the need for dual signaling effects by intracellular FGF-2 and receptor-mediated signaling for inducing transformation. In addition, soft agar colony formation by clones expressing low quantities of transduced FGF-2, which were not exported to the extracellular space, resulted in significantly fewer colonies than control vector-transduced NIH-3T3 cells, suggesting an inhibitory effect on transformed behavior by intracellular signaling alone [103]. These experiments suggest that induction of Bcl-2 and prevention of apoptosis are the results of the expression of low levels of intracellular FGF-2, which are not exported. However, dual signaling by receptor-mediated exported FGF-2 in conjunction with intracellular signaling is necessary for transformation [103]. Of note, the observed decrease in soft agar colony formation by low levels of intracellular FGF-2 in comparison to control vector-transduced cells suggests a differentiation effect and inhibition of transformation by purely intracellular FGF-2 [103].

Overall, the data support a general model where both intranuclear and receptor-mediated signaling together are necessary for proliferation and transformation. Intracellular signaling, both cytoplasmic and nuclear, without receptor-mediated signaling may promote only proliferation and survival signaling. However, nuclear signaling alone may inhibit proliferation, motility and transformation (Figure 1).

### 3.3. Extracellular Export

FGFR-mediated canonical signaling and internalized FGF/FGFR direct signaling are further complemented by the intracellular expression of FGF species, their direct intracellular signaling and export, enabling them to undertake autocrine and paracrine FGFR-mediated signaling once they are external to the cell. Most FGF family members, except for FGF-1 and FGF-2, have hydrophobic signal peptides and are exported after expression through the endoplasmic reticulum and the Golgi apparatus [191,192,193,194]. FGF-11-FGF-14 also lack signal peptides and remain exclusively intracellular [168]. FGF-1 and FGF-2 do not have signal peptides, but are nevertheless exported by other, non-classical mechanisms [195,196,197,198]. If a signal peptide is added to FGF-1, it becomes a potent oncogene [199]. This suggests that the loss of the signal peptide may be part of a potential evolutionary process from its presence in Caenorhabditis elegans and Drosophila, an adaptive loss that eliminated its strong, unregulated oncogenic potential during the reproductive life segment of adult mammals [193,195,200].

Instead of using a signal peptide, FGF-1 has developed alternate mechanisms. The export of FGF-1 from the cytoplasm requires its monomer to dimerize through a disulfide bond [201,202,203] and undergo appropriate folding [204]. The FGF-1 dimer is exported through its non-covalent binding to a complex consisting of the 40 kDa (p40) form of the docking protein Synaptotagmin 1 (Syt1), the small calcium-binding protein S100A13, SK1, an enzyme responsible for phosphorylation of sphingosine and production of a proangiogenic mediator, sphingosine 1-phosphate (S1P) [205], and annexin II [193,200,206,207,208]. The efficiency of this export complex is enhanced by copper [193]. The Notch pathway, acting through the mammalian transcription repressor RBP (CBF1) and its partner Mastermind-Like (MAML), suppresses the transcription of FGF-1 and sphingosine kinase 1 (SphK1), an obligate component of the FGF-1 export pathway [53]. CBF1 suppresses the S100A13-mediated release of transduced FGF-1 and the FGFR-1-mediated autocrine induction of tumorigenicity in vitro and in an in vivo murine model [53]. FGF-1 requires stress for release from cells, such as heat shock [209], hypoxia [210], or low serum [211,212]. In the case of FGF-3, once it is synthesized in the cytoplasm, it accumulates in an immature form in the Golgi complex, from where it is slowly released into the ECM [213]. The extracellular export and nuclear localization of the CUG-initiated FGF-3 occur in similar proportions [153,157]. An FGF-3 species engineered to eliminate its extracellular export sequences, forcing it to only localize to the nucleus, and specifically in the nucleolus, inhibited DNA synthesis and cell proliferation [179].

Intracytoplasmic FGF-2 is also not incorporated into transport vesicles but, rather, is directly translocated extracellularly across plasma membranes [214,215]. Both LMW and HMW FGFs are exported by MCF-7 BC cells transduced with vectors expressing them [103]. Reports show that HMW FGF-2, mainly 22, 22.5 and 24 kD species, are exported through HSP under stress by BC cells [165], although they are not exported in other cell types; instead, they are primarily internalized into the nucleus through processes involving nuclear localization sequences [136,143,144]. FGF-2 is recruited to the inner leaflet of the plasma membrane, sequentially with an obligate Tec kinase-dependent FGF-2 tyrosine 82 phosphorylation, and appropriate folding for phosphatidylinositol 4,5-bisphosphate (PI(4,5)P) 2-dependent FGF-2 oligomerization and membrane pore formation [216,217,218,219]. The complex directly traverses the plasma membrane to access the extracellular space in an ATP-independent manner [214,215]. The multidrug resistance-associated protein and the Na^+^-K^+^-ATPase are also involved in the process [220,221]. Once crossing to the extracellular space, FGF-2 has an obligatory dependence on extracellular trapping by HSPG membrane-proximal binding sites [198,215,219,222]. Extracellular FGFs are deposited and stored on extracellular HSPGs on the surfaces of cultured bone marrow stromal cells [223], and HSPG-FGF-2 complexes can be released from bone marrow stromal monolayers by Phosphatidylinositol-Specific Phospholipase C (PI-PLC) [224]. HSPGs protect exported FGFs from proteolytic degradation and thermal denaturation [118]. Unlike FGF-1, FGF-2 does not require stress for its extracellular release [221,225].

### 3.4. Key Takeaways from Section 3

1. Microenvironmental LMW FGF binds FGFRs to induce proliferation and survival.
  - Membrane FGF/FGFR complexes initiate canonical signal transduction.
  - FGFs can bind integrins to initiate signaling to promote cell cycle progression.
  - FGF/FGFR complexes internalize, and initiate direct cytoplasmic and nuclear signaling.
2. Exported autocrine LMW and HMW compete in FGFR binding to induce proliferation and survival. Many FGFR species are exported without a signal sequence.
  - Membrane FGF/FGFR complexes initiate canonical signal transduction and then internalize. HWM FGF species initiate FGFR signaling less efficiently than LMW species, and can competitively inhibit canonical proliferative signaling by LMW isoforms.
3. LMW FGF can induce FGFR-mediated growth inhibition and dormancy in conjunction with fibronectin-mediated integrin signaling.
4. LMW FGF expression and cytoplasmic localization can induce cell survival through direct cytoplasmic signaling before being exported.
5. HMW FGF expression in stressed non-transformed or cancer cells translocates to the nucleus through alternate translation initiation- or splice-generated NLS and induces growth inhibition by nuclear signaling combined with hormone receptor (HR) signaling.
6. HMW FGF expression, nuclear localization and signaling cooperate with FGF/FGFR canonical signaling and FGF/FGFR internalization to induce transformation, proliferation, and survival. Key points are graphically summarized in Figure 1 and Figure 2.

## 4. Implication of FGF Expression in BC Progression

Dual effects of FGF signaling have been reported in BC, where investigators recognizede that FGF-2 is both stimulatory and inhibitory, depending on cell type, experimental design, context [226], and paracrine and adhesion-mediated microenvironment signaling [39,40,47,101]. The historical mechanistic studies reviewed above support a model where FGFR-mediated canonical signaling, FGF/FGFR internalization and non-canonical signaling combine with intracellular signaling by newly expressed FGF to promote cancer progression, while non-canonical signaling by intracellular FGF expression independently inhibits cancer progression. This section presents data in BC supporting this hypothesis. The data are organized by the BC cell types, the level of support and limitations of evidence. Due to the variety of approaches, complexity of the models and data obtained, most individual studies generate data to support only parts of the hypothesis with varying impact. A consolidated analysis of the individual pieces of evidence supports the hypothesis.

### 4.1. Data Supporting Pro-Tumorigenic Effects of FGF Expression

Invasive ductal carcinoma of the breast is allocated to broad categories of slow-growing luminal A, ER/+/PR+/Her2− cells, faster-growing luminal B, ER+, often PR− cells which can be Her2+ or Her2−, the Her2+ cell category, and the highly aggressive TNBC, ER−/PR−, Her2− cells. The percentage of cancer stem cells is very low, less than 1% in the Luminal A cancers and increases progressively and highly variably, to up to a third or more in TNBCs [227,228]. Cancer-promoting effects of the expression of FGF family members were observed in most BC subtypes [229]. Results, outlined and referenced according to FGF family members in the sections below, demonstrated effects in HR+, in TNBC, and in multiple BC types. Generally, the pro-malignant effects were reported in the context of combined signaling by autocrine or paracrine FGFR activation, when data were available.

#### 4.1.1. Multiple BC Molecular Subtypes

FGF family members from all three categories, canonical, endocrine and intracellular, have demonstrated associations between FGF expression and pro-malignant effects. The studies outlined below delineate data from each FGF category on their associations with cancer effects.

From the canonical category, FGF-1, -2, -4, -7, and -16 have been investigated for their association with pro-malignant effects in multiple BC types. FGF-1 immunohistochemical staining was present in the majority of human BC tissues analyzed from both ER/PR+ and ER/PR− BC from 80 patients, both in epithelial cells and in stromal cells, particularly around malignant cells. FGF-1 also stained positive in non-malignant tissue epithelial cells but not in the stroma [229]. Both malignant and non-malignant epithelial cells contained FGFR3 by immunohistochemistry, but FGFR3, which was confirmed to be FGFR3b, the epithelial, pro-proliferative splice variant by reverse transcriptase (RT)-PCR, was predominantly nuclear in malignant cells, whereas it stained positive in both the cytoplasm and nucleus in epithelial cells in normal breast tissue [229]. Myoepithelial cells and stroma stained negative for FGFR3 [229]. These data support a necessary role for FGFR3b isoform-mediated signaling and nuclear translocation in promoting and sustaining the malignant phenotype, and the need for combined receptor-mediated and intracellular FGF signaling to promote malignant behavior. The primary limitations of the data are that they are associations and limited to RNA expression.

Variables such as gene amplification, mutations, exon modifications, altered mRNA and protein expression levels, and epigenetic modifications, such as DNA methylation, contribute significantly to BC carcinogenesis [230,231,232]. Specifically, expression of the m^6^A reader YTH N6-methyladenosine RNA binding protein 3 (YTHDF3) is abnormally elevated in various BC subtypes [232]. YTHDF3 is associated with a poor prognosis and plays key roles in BC cell self-renewal, cell growth, migration, invasion and metastasis [232]. The effects are mediated through the maintenance of FGF-2 translation by inhibiting FGF-2 transcript degradation mediated by binding to methylated coding sequences [232]. Elevated levels of YTHDF3 in BC tissue immunohistochemical stains are present in cancer cells and the microenvironment [232]. These investigations are also associations only and lack receptor expression and signaling data.

FGF-4 levels were higher in tumors and adjacent normal tissue and were associated with larger tumor size, lymph node metastasis and short survival time in both ER/PR+ and TNBC patients and with malignant behavior in vitro and in vivo in ER/PR+ and TNBC cell lines. Direct targeting of FGF-4 using miR-511 to decrease its levels abrogated these malignant phenotypes, which were subsequently restored with enforced expression of FGF-4 [233]. The patient data demonstrate associations but lack receptor information, while the preclinical interventional data demonstrate the necessity of FGF-4 signaling for malignant phenotypes in a cell line but also lack FGFR data.

FGF-7 mRNA and protein levels are high in ER+ and ER- BC cell lines in vitro and in vivo in xenografts [234], and induce proliferation, migration, invasion, tumor growth and inhibit apoptosis [235]. Inhibition of FGF-7 with miR-1236-3p [234] or the isoquinoline alkaloid Berberine (BBR) [235] diminishes the malignant phenotype. BBR inhibits BC cell proliferation, invasion, and migration in vitro, tumor growth in vivo, and induces apoptosis by downregulating the high levels of FGF-7 in BC through downregulation of methyltransferase-like 3 (METTL3) and its effects on m6A-mediated stabilization of FGF-7 mRNA [235]. Immunohistochemical staining of the tumors suggested there was positive staining for FGF-7 in the TME [235]. Indeed, FGF-7 is expressed and exported by cells of the TME as well as by the BC cells, superimposing receptor-mediated effects on its intracellular effects [236,237,238,239]. These studies demonstrate the necessity of FGF-7 expression in vitro and in vivo but are only preclinical and lack receptor data or definitive evidence of dual signaling.

FGF-16 mRNA and immunohistochemical staining demonstrated elevated expression in 26 human tumor samples with a wide range of combinations of HR+/−/Her2+/− molecular subtypes compared to adjacent normal tissue [240]. These studies provide association support for FGF-16 expression and malignancy. FGF-16 induced expression of transcriptional regulators ZEB1 and SNAIL, and mesenchymal markers Vimentin and N-Cadherin [240]. It also induced ECM-degrading and remodeling enzymes, including MMP2 in MDA-MB-231 and MCF10A cells, and regulated a set of genes involved in the remodeling of the ECM [240]. These programmatic changes in expression were induced by receptor-mediated FGF-16 signaling through induction of aerobic glycolysis by robust expression of 6-phosphofructo-2-kinase/fructose-2, 6-bisphosphatase 4 (PFKFB4) and Glucose Transporter (GLUT)3, mediated by MAPK signaling [240]. Enforced expression of FGF-16 induced greater malignant traits in HR− tumor cells in vitro and in an in vivo model [240]. These studies demonstrate sufficiency and provide mechanistic support for the malignant effects of FGF-16, but are preclinical and do not address dual signaling.

From the endocrine category, FGF-19 demonstrated a pro-malignant association. A study examined the levels of FGF-19 in 73 breast cancer patients in pre- and post-neoadjuvant paclitaxel chemotherapy samples by IHC staining and showed that paclitaxel increased the levels of FGF-19 in all categories of BC patient tumors, and in patients who developed metastases post-paclitaxel treatment. A Kaplan–Meier plot showed that higher FGF-19 expression levels were strongly associated with lower RFS in BC patients [12]. In preclinical models, paclitaxel promoted BC progression by increasing the expression of FGF-19 through ATF4 via a stress response, increased export of FGF-4 into the medium and higher levels of phospho-FGFR4, suggesting autocrine signaling, and promoted BC cell migration and in vivo tumor progression in ER+ and TNBC cells [12]. These associations support dual signaling through FGFR4.

From the intracellular category, FGF-11 and -13 showed an association with malignant effects. Data from expression profiling of four different genome-wide microarray platforms on enriched malignant neoplastic epithelial cells from freshly isolated 50 primary BCs and luminal and myoepithelial cells were compared to cells from primary human epithelial cell cultures from mammoreductions [170]. The study concluded that FGF-11 is expressed at higher levels in enriched malignant cells compared to normal epithelial cells, albeit both being expressed at low levels [170]. Another study also concluded that FGF-11 is expressed at a significantly higher rate in tumors than benign tissue in both ER+ and ER- BC [171]. However, median OS and PFS were not statistically different between groups with different levels of FGF-11 expression [171]. Gene array analyses did not reveal differences in expression in any of the other FGF family members between benign and malignant cells, but FGFR1 and FGFR4 were uniquely expressed in malignant cells and not in normal epithelial cells. The expression of FGFRs and the fact that the intracellular category of FGFs do not signal through receptors further support the thesis that additional FGFR-mediated signaling by other FGF family members is needed for the malignant effects of intracellular FGF protein expression.

Preclinical studies demonstrate variable pro-tumorigenic effects of FGF-13. Aggressively metastatic TNBC MDA-MB-231_HM cells express higher levels of FGF-13 mRNA than non-metastatic parental cells [169]. FGF-13 is necessary for MDA-MB-231_HM cell in vitro colony formation and for in vivo lung and liver metastasis from xenografts, but has only a negligible impact on primary tumor growth and no impact on colony formation of MCF-7 and MDA-MB-361 BC cell lines [169]. Association studies of patient tumors also demonstrated variable effects. Higher FGF-13 mRNA expression was associated with worse relapse-free survival in luminal A and basal-like human BCs than lower FGF-13 expression analyzed from 3951 fresh-frozen samples and adjacent uninvolved normal breast from repositories [169]. However, FGF-13 was not higher in primary tumors compared to normal breast, and was not associated with other clinical variables [169]. These studies did not provide any receptor or molecular context.

#### 4.1.2. HR+ BC

The expression of canonical FGF category members FGF-1, -3, -4, -5, -8, -10, -18 and -20 has been reported to promote procarcinogenic effects of HR+ BCs. Overexpression of FGF-3 protein in spontaneous breast tumors of MMTV-infected albino mice, characterized by a progression from ER+ to ER- as the disease advances, played a significant role in BC formation through export and autocrine stimulation through FGFR1, STAT3 and Akt signaling [69]. These studies provide proof of dual signaling in procarcinogenic effects but only in a preclinical model.

Four percent of MMTV infection-induced CH3 mammary tumors had activation of FGF-10 expression by MMTV insertion [241]. Immortalized ER+ HC11 mammary epithelial cells from spontaneous mutations in both p53 alleles were transduced with an FGF-10 vector and proliferated significantly faster than control cells in vitro [241]. FGF-10-transduced CH3 cells formed invasive, larger, more frequent (6/6 vs. 4/6) and more vascularized CK8+ better-differentiated duct-forming tumors in BALB/c mice than controls, but were not highly proliferative in vivo [241]. The study authors suggested that FGF-10 can act as an oncogene in that system, likely due to cooperation with mutant p53 [241]. However, while low variable levels of FGF-10 mRNA were found in over 90% of 50 primary human BCs of unspecified variable hormone receptor expression, high levels were only found in about 10% of cancers randomly selected for high [>50%] epithelial/stromal ratios and in 3 of 5 normal breast tissue samples from reduction mammoplasties [241]. In fact, FGF-10 functions in mammary duct development in a paracrine manner, exported by stroma acting through FGFR3b, suggesting that receptor-mediated signaling, in addition to the low levels of expression, may be necessary for malignant effects. Indeed, human samples analyzed were bulk surgical samples containing cancer and stromal cells [241]. These studies support associations of FGF-10 and FGFR3b in dual signaling in relatively small sample sizes without actual signaling mechanism data.

Analysis of the amplification of the 11q13.3 locus, observed in a number of BCs from 156 BC samples from the cBioPortal platform (TCGA), demonstrated amplification of locus-contained genes coding for CCND1, FGF-3, FGF-4, FGF-19 and Fas-associated death domain (FADD) [13]. This amplification was inversely correlated with tumor infiltration by CD4+ T cells and dendritic cells (*p* < 0.05), and CCND1, FGF-4 and FADD mRNA expression were closely correlated with shorter recurrence-free survival in patients with BC (*p* < 0.05) [13]. The limitations of the study relate to the multiple genes regulated by the amplification and the lack of FGFR and signaling data.

A study with total RNA samples isolated from 5 specimens each from total breast tissue at the time of surgery obtained from 61 BC and 47 normal patients, which were reverse transcribed using RT-PCR and analyzed by microarray analysis, demonstrated that cancer samples had higher levels of expression of FGF-5 (arbitrary units 5.466 ± 0.361) compared to normal samples (arbitrary units 5.193 ± 0.201) (*p* < 0.0001) [7]. Differences in patient features were not statistically significant. FGF-5 expression in the low-expression group was correlated with better clinical characteristics, including tumor size, grade, estrogen receptors, clinical risk category, hormone and HER2 receptor expression, lymph node involvement and Veridex 76-gene signature, and a better DFS [7]. The cell source of FGF-5 expression from the bulk tumor samples was not determined. In addition, the studies demonstrate only associations, have small sample sizes, and lack protein and FGFR expression and signaling data.

In an unsupervised cluster analysis of 98 breast tumors, the expression pattern of 70 genes in 78 samples of RNA extracted from snap-frozen total primary tumor samples revealed a cluster of genes with the greatest association with poor prognosis and distant metastases [78]. Among these, the downregulation of FGF-18 was highly correlated with the absence of distant metastases for more than 5 years [78]. A majority of the HR+ patients were grouped into the cluster of genes with a good prognosis. However, the effect of FGF-18 signaling in the context of the prognostic gene cluster cannot be evaluated beyond the given data, and the study documents only an association.

FGF-1 transfected MCF-7 cells implanted into ovariectomized nude mice with and without estrogen supplementation formed large, vascularized tumors, but lymph node and pulmonary micrometastases were only found in mice that did not receive estrogen supplementation. In vitro, FGF-1 overexpressing cells exhibited increased anchorage-independent growth and shortened doubling time in estrogen-depleted medium compared with estrogen-replete medium, becoming estrogen-independent [242]. From the data, the authors suggested that FGF signaling may be important in the transition of BC cells from hormone-dependent to hormone-independent, and from non-metastatic to metastatic capabilities [242]. Enforced expression of FGF-1 and FGF-4 in MCF-7 cells induced growing xenograft tumors, with DNA replication being closely associated with tumor growth [243]. Mouse tumors had increased edge-associated intratumor microvessels, but not stromal microvessels, as compared with tumor nodules collected under growth-inhibiting conditions [243]. These observations supported an autocrine effect, although there was no discussion of export of FGF-1 by MCF-7 cells in the manuscript [243]. These fast-growing tumors that formed without estrogen supplementation had higher blood flow, higher tumor pO2, which was preserved even in larger tumors, and formed metastases in LNs, lung, brain, kidney, perirenal fatty tissues, liver, spleen, retroperitoneum, heart, and gallbladder [244,245]. These results suggested that tumor angiogenesis, through a paracrine process, was also a key factor in the tumor promotion mechanisms by FGF-1 and FGF-4 [243,244], in addition to FGF-1-mediated direct cancer cell effects [243]. While the evidence is highly supportive of a dual effect, its primary limitation is that it is only preclinical, using cell lines.

Alternatively spliced FGF-8b, a ligand for mesenchymally spliced FGFR1c, FGFR2c and FGFR4, was shown to be the most transforming isoform in MCF-7 cells [246]. Enforced expression of FGF-8b induced aggressive malignant phenotypes in vitro, including increased anchorage-independent growth, invasion due to upregulated matrix metalloproteinase 9 secretion, and in vivo in mouse xenografts, including highly vascular tumor growth, supporting the paracrine and autocrine effects of FGF-8b in MCF-7 cells [247]. This data provides strong evidence for dual signaling, but it is limited to a cell line.

In the endocrine category, FGF-19 and FGF-23 expression have been associated with malignant effects. FGF-19 is expressed at high levels, is exported and signals in an autocrine manner through FGFR4 in ER/PR+ BC, and is associated with aggressive malignant behavior and poor OS and RFS of breast cancer patients [8]. Enforced expression of FGF-19 also promotes malignant functions in both ER+ and ER- BC xenograft tumors, where its functions are mediated through FGFR4-AKT signaling [8]. The clinical studies are associations and the enforced expression studies that demonstrate FGFR4 signaling are convincing in cell lines but are preclinical models that do not necessarily reflect the complexity of human cancers.

A Mendelian randomization analysis based on a genome-wide association study (GWAS) of patients of European ancestry demonstrated that FGF-20 showed a small genetically protective effect on overall BC and ER+ BC, FGF-4 was associated with a small increased genetic risk of overall BC, FGF-1 and FGF-7 were associated with small increased genetic risks of ER+ BC, and FGF-23 promoted the effects in ER− BC [14]. In the converse analyses, ER+ BCs were associated with elevated FGF-7 and decreased levels of FGFR2 [14]. Once again, caution must be emphasized in generalizations derived from GWAS on linking phenotypic associations with the expression of FGFs in cancer epithelial cells, since the source of the growth factor RNA from tumor samples is most likely not exclusively from cancer cells.

#### 4.1.3. TNBC

Canonical FGF category members FGF-2, -5, -9, and -18 had associations with pro-malignant effects in TNBC. FGF-2 and S100A4 expression are higher and positively correlated in TNBC with respect to non-TNBC patients when analyzing the Cancer Genome Atlas (TCGA) dataset [248]. Exogenous FGF-2 induces upregulation and secretion of S100A4 via FGFR1, along with ERK1/2-AKT-c-Rel activation, and activates vascular endothelial cell and CAF migration [248].

In a panel of 31 BC cell lines, 62% of basal-like breast cancers expressed FGF-2, as assessed by immunohistochemistry, compared with 5% of non-basal breast cancers [249]. FGF-2 expression promoted cell growth in vitro and xenograft growth in vivo, but once again, driven by autocrine receptor-mediated growth signaling [249]. The FGFR inhibitor PD173074 significantly blocked anchorage-independent growth while having only a modest effect on 2D growth selectively in TNBC cells [249].

In a positive feedback loop between TNBC cells and stromal cells, MEKK1 in non-transformed fibroblasts promoted CCL5 expression and promoted tumor cell invasion through the GPCR CCR5 receptor in MDA-MB-231 TNBC cells, which, in turn, synthesized and exported FGF-5 and positive feedback activated MEKK effectors ERK1/2 and NFκB that induced CCL5 expression in fibroblasts [250].

FGF-5 expression analyzed by qPCR was significantly upregulated in TNBC tissues and cells as compared with corresponding paracancerous normal breast tissue from 57 patients [251]. In BC cell lines, FGF-5 expression is associated with cell proliferation, migration, invasion, tube formation, and diminished apoptosis [251]. FGF-5 overexpression in bulk tumors was also found in a large panel of upregulated gene expression signature arrays from a bone-homing TNBC MDA MB 231 BC cell line [15].

In the tumor microenvironment, CAFs significantly promote the proliferation, invasion, and migration in vitro and in xenograft transplants of TNBC cells by upregulating FGF-9 and NOVA1, underscoring the outsized impact of the TME and CAFs on tumor progression [252].

MDA-MB-231 human TNBC cells express FGF-18, and both enforced expression, siRNA knockdown, as well as exogenous FGF-18 had FGFR-mediated effects on gene expression and ERK activation in vitro [80]. FGF-18 promoted proliferation through FGFR/ERK/c-Myc signaling, EMT program activation and cell migration, invasion and increased xenograft tumor growth [80]. FGF-18 knockdown reversed the signaling and gene expression activation, causing loss/reduction of p-ERK, c-Myc, N-cadherin, vimentin and Snail 1 protein in MD+A-MB-231 cells [80]. These data further support the roles of paracrine or autocrine receptor-mediated signaling combined with intracellular FGF expression signaling in promoting cancer progression in cancer cells.

The endocrine category members Fgf-19, -21 and -23 also had associations with pro-malignant effects in TNBC. FGF-23 was associated with pro-cancerous effects in ER- BC in a genome-wide association study (GWAS) [14] and was implicated in the formation of bone metastases [11]. Higher expression of Fgf-19, Fgf-21 and Fgf-23, canonical FGF-1, and reduced expression of canonical secreted FGF-2 were measured by qRT-PCR in the MDA-231-BoM-1833 TNBC cell line established from bone metastases of nude mice as compared with MCF-10A immortalized non-transformed mammary epithelial cells [253]. Signaling by these FGF family members through FGFR1 in osteoclasts induced osteoclast differentiation and activity in vitro and osteoclast activity and bone destruction in vivo in a mouse model [253].

Although all of the references in the above sections present data supporting the pro-malignant effects of FGF expression in BC, references [8,12,69,80,247,249] provide the most compelling partial evidence, each with its own limitations, that support the hypothesis put forth in this review that dual FGFR-mediated classical signaling combined with intracellular expression of FGF family members induces pro-carcinogenic effects in BC.

#### 4.1.4. FGFR Role in Pro-Tumorigenic and Treatment Resistance Effects in BC

Mutations, amplification and overexpression of FGFRs drive malignancy through ligand-independent activation of multiple signaling pathways [3,254] and increase the risk of metastasis [255]. FGFR1 and FGFR2 are the most common genetic mutations in BC, with 10–20% of BCs, particularly ER/PR+ and Her2− cancers, having FGFR1 amplifications, which correlate with high histopathological grade and high proliferation [256], distant metastases [257], brain metastases [258] and poor prognosis [255,259,260,261]. In an immunohistochemistry staining of 426 tumor samples and adjacent tissue from archived surgical tissue, FGFR1 levels were expressed at higher levels in tumors than in tumor-adjacent tissues, and high FGFR1 expression in tumor tissues was associated with less favorable histological grade and high Ki67 proliferative index tumors of Luminal B-like subtype [262]. FGFR2 was also drastically increased [263,264] and had a consistently strong association with BC [37]. Overexpression of FGFR4 also predicted unfavorable OS in an analysis of online databases [265].

The activation status of FGFR2 is relevant to its prognostic effects. Cytoplasmic and nuclear FGFR2 were expressed in 64.8% and 56.8% of BC patients, respectively, and cytoplasmic FGFR2 expression and lymph node metastasis were independent negative prognostic factors for both disease-free survival (DFS) and OS by multivariate analysis [266]. TNM stage and tumor size were positively correlated with FGFR2 expression. The risk ratios for cytoplasmic and nuclear FGFR2 expression were 2.84 and 2.34, respectively, and the overall risk ratio for FGFR2 overexpression was 2.79 in the Cox Proportional Hazards Model. Kaplan–Meier DFS and OS were significantly worse for patients with high staining scores for cytoplasmic (*p* = 0.007 and *p* = 0.009) and nuclear FGFR2 (*p* = 0.014 and *p* = 0.017) in the 125 patients in the study [266]. Thus, not only is it necessary to identify the expression of FGFRs specifically in BC cells from tumor tissue in assessing their contribution to the outcome of BC, but, as these results suggest, it is important to obtain data that reflect their activation and internalization status. The overall data support the combined cancer-promoting effects of intracellular FGF and FGFR signaling.

Constitutively active FGFR signaling can induce treatment resistance, potentially by effectively modifying the characteristics of the BC cells into cancer stem cells. Tumors with upregulated FGFR1 expression and poor prognosis and cancer characteristics had upregulated Sonic Hedgehog (SHH) pathway genes, including its transcriptional target and effector gene Glioma-associated Oncogene Homolog 1 (GLI1) [267]. In a TNBC cell line, FGFR1 and GLI1 had strong positive feedback mechanisms and were directly responsible for cell proliferation, motility, and invasion, suggestive of a causal role of their crosstalk in metastasis [267]. FGFR2 overexpression in cells induces expression of Runt-Related Transcription Factor (RUNX)2, cell proliferation, metastasis and increased aggressiveness [268]. Association of FGFR1c, the FGFR1 splice variant responsible for mesenchymal programming, along with its type 1 transmembrane glycoprotein, a high-binding-affinity partner, Nonmetastatic Melanoma Protein B (GPNMB), overexpressed in TNBCs in GPNMB^high^/FGFR1^high^ cancers, was associated with shorter RFS, in vitro malignant phenotypes, Akt phosphorylation and EMT in TNBC cells [269]. Effectively, the expression of these stem cell signaling pathways likely promotes a cancer stem cell phenotype, which contributes to therapeutic resistance of BC cells with constitutive FGFR signaling. In fact, chronic FGF-2 treatment can convert induced pluripotent stem cells (iPSCs) into cancer stem cells [270].

However, FGFR signaling alone is not the primary determinant of the exogenous pro-malignant effect of their extracellular signaling pathways; instead, signaling from the TME may have an even greater impact. Evidence strongly suggests that the tumor microenvironment provides significant context into BC response to FGF family members [271,272]. The profile of the TME modulates the prognostic significance of FGFR2 expression, and differences in stromal gene expression patterns may account for inconsistencies in prognosis of FGFR2 expression [271]. The ECM acts as a reservoir and presenting instrument for growth factors to adjacent epithelial cancer cells, with FGF-2 as a prime factor driving ER+ BC malignancy that generates increased ERK-dependent, estrogen-independent ER signaling and endocrine therapy resistance [76]. CAFs in the TME express and export FGFR ligands FGF-2, FGF-5, FGF-17 [272] and FGF-9 (252), but their poor prognostic effects are primarily correlated with upregulation of Alpha-Smooth Muscle Actin (αSMA), Fibroblast-Specific Protein 1 (S100A4/FSP-1), Platelet-Derived Growth Factor Receptor (PDGFR), Podoplanin (PDPN), Hepatoma-Derived Growth Factor (HDGF), PDGF, C-X-C Motif Chemokine Ligand (CXCL)8, CCL5, CCL2, Interleukin (IL)-6, Hedgehog (HH) and EGF, with the effects of other markers being variable [272], and are turned into active promoters of cancer progression by the malignant epithelial cells [273]. FGFR2 participates in regulating degradation and organization of the ECM and retinoic acid signaling, and its effects are closely dependent on CXCL8, CD44, MMP9, and BMP7 [265] and the prognostic impact of FGFR2 depends on high expression of the stromal genes delineated [271]. These and other variables need to be considered in evaluating FGFR signaling pathways as therapeutic targets in BC.

#### 4.1.5. Key Takeaways from the Data on the Cancer-Promoting Effects of FGF Expression

The data in this outline of studies suggest an association of FGF protein expression with promotion of malignant effects in BC. However, there are a number of confounding variables, outlined below, that limit definitive conclusions.

1. Expression of canonical FGF family members FGF-1, -2, -3, -4, -5, -7, -9, -10, -16 and -18, endocrine FGF family members FGF-19, -21 and -23 and intracellular FGF family members FGF-11 and -13 were associated with pro-malignant behavior of human tumors and BC cell lines.
2. Enforced expression of FGF-1, -2, -4, -8b, -10 and -18 induced more malignant behavior in BC cell lines in vitro and in vivo in animal studies. These FGF proteins were exported and engaged in FGFR-mediated autocrine co-signaling.
3. Expression of FGFRs, type, splice variant, polymorphism, mutations, amplifications, cellular localizations and receptor-mediated co-signaling contributed to the effects.
4. Hormone receptors: more malignant behavior was documented in both ER/PR+ and ER/PR− tumors and cell lines expressing FGF family members.
5. Source of exogenous FGF initiating FGFR-mediated signaling: cancer-associated fibroblasts, stromal deposits and non-fibroblast cells, systemic sources, and autocrine signaling by cancer cell-secreted FGFs. TME characteristics contribute significantly to BC outcome.
6. Cellular compartments: FGFR co-staining and nuclear localization correlated with the data that intracellular expression combined with receptor signaling, internalization and intracellular signaling are necessary for a pro-malignant effect.
7. Analytic scale: bulk tumor qrtPCs, gene array analysis, proteomic and phosphoproteomic analysis, and nipple fluid enzyme-linked immunoassay (ELISA) all provided expression data from the combined sources of the cell content of the sample and represented the bulk of the association studies.
  Immunohistochemistry and single-cell rtPCR provided single-cell expression content. These represented some of the association studies and many of the causative pre-clinical studies. Some bulk tumor and cell culture preclinical studies also included Western blot and Northern blot analysis.
8. Survival analyses from massive public datasets have very large confidence intervals. Analyses do not account for treatment-related variables. Predictions based on any one variable are affected by hundreds of other variables intrinsic to any specific scenario.
9. There were significant limitations to proving the dual signaling hypothesis in human breast tumors in all of the studies. The following outline highlights some of them.
  a. Bulk analyses do not identify the cellular and subcellular sources or initiation variants of FGF family member expression and export. Most studies do not assess or identify FGFR type expression, splice variants, polymorphisms, mutations, or amplifications in the cancer cells or in the stromal cells.
  b. Gene expression analyses documenting hundreds to thousands of upregulated and downregulated mRNAs do not provide context for the importance of regulation of a particular or several FGF family members.
  c. Tumor specimens vary by size of the tumor, origin and location of specimen, whether it is internal or at the edge of the tumor, presence of structural proteins, cellular and vascular contents that vary with location within a tumor, from tumor to tumor in a patient and between patients.
  d. Parallel signaling by other growth factors and structural protein receptors, non-canonical FGFR activation, oncogene drivers or mutated p53, as well as immune system co-modulation and interaction with cancer cells provide confounding effects that may enhance or interfere with the FGF family protein-mediated signaling.

Overall, the effects of FGF are masked and affected by these variables. The design of experiments to identify and characterize expressed FGFRs, knockout specific FGF receptors, identify parallel signaling, control experimental conditions, or design clinical trials must be guided by considerations of the potential confounding effects. Even then, they will extract population-averaged data with significant confounding variables. Human tumor expression data only provided associations. Some preclinical studies provided proof of need for dual intracellular and receptor signaling for pro-cancer effects but did not demonstrate all the factors of molecular signaling outlined in historic studies that generated the dual signaling hypothesis. Also, these models do not faithfully mimic human BCs. Table 3 outlines the modes of experimental support, the components of the dual signaling hypothesis partially supported by the studies cited, and their key limitations.

### 4.2. Impact of the Immune System

In a small study, progression from ductal carcinoma in situ (DCIS) to invasive ductal carcinoma (IDC) was associated, exclusively in Luminal A tumors, with increased infiltration by CD8+ T cells, CD68+ macrophages, and FOXP3+ staining, indicating an immune-suppressive TME [274]. FGFR2 expression positively correlated with these immune markers, suggesting a synergistic relationship between FGFR2 and ER signaling and immune evasion and tumor progression [274]. As noted above, amplification of a locus-containing genes coding for FGF-3, FGF-4, and FGF-19 was inversely correlated with tumor infiltration by CD4+ T cells and dendritic cells, and a correlation of FGF-4 expression with shorter recurrence-free survival in BC [13]. In an analysis of public genomic datasets and copy number variations in BC tumors, regulatory T-cells were associated with the basal-like subtype and type 2 T-helper cells were associated with the HER2+ and the luminal subtype [275]. However, the most relevant processes included the ERBB2 and FGFR signaling pathways, where amplifications of FGFR genes strongly correlated with the presence of type 2 T helper cells [275].

Interferon (IFN)γ from T-cells in the TME promotes tumor FGF-2 signaling, suppresses pyruvate kinase M2 (PKM2) activity, and decreases NAD+ and Sirtuin 1 (SIRT1), which results in reduced β-catenin deacetylation, enhanced β-catenin acetylation, and reprogrammed tumor stemness. Patients treated with immune checkpoint inhibitors can have distinctly opposite responses, either a complete response or a resultant hyperprogressive disease [276]. The difference does not appear to be based on immune exclusion based on CD8+ T cell infiltration and IFNγ gene signatures, which are similar in the two circumstances, but rather on elevated tumoral FGF-2 and β-catenin signaling, underscoring the crosstalk of FGF-2 oncogenic signaling with core immunogenic and metabolic pathways [276,277]. Indeed, in a genomic data set of 545 immune checkpoint blockade (ICB)-treated patients, patients with no FGF-2 and FGFR genomic amplification had significantly longer PFS than patients with FGF-2/FGFR amplifications. However, in a cohort of patients untreated with ICB, FGF-2/FGFR genomic amplification was not associated with prognosis, thus suggesting that FGF-2/FGFR genomic amplification was a predictive biomarker of immunotherapy resistance [277].

In TNBC, FGF-4 drives cancer progression by modulating macrophage polarization through the IL-6/STAT3 signaling axis, and promotes immune suppression and TME remodeling. In an analysis of a variety of molecular and functional datasets, IL-6/STAT3 emerged as a key pathway [278]. In an in vitro model, FGF-4 activated IL-6/STAT3 signaling, induced macrophage polarization to an M2 phenotype with elevated IL-10/TGFβ secretion, and suppressed T cell proliferation [278]. M2 macrophage-conditioned medium enhanced TNBC cell aggressiveness. In orthotopic TNBC tumor models, increased FGF-4 expression caused higher tumor volumes and enhanced CD8+ T-cell infiltration [278]. Enrichment in M2-like macrophages was also found in FGF-2-rich areas in primary tumors, but these were accompanied by diminished numbers of CD8+ T cells and B cells [279]. Indeed, blocking FGFR signaling caused a marked increase in T-cell infiltration and suppressed tumor growth in an immunocompetent mouse model of TNBC [280]. The effect was mediated by inhibition of CAF proliferation through ERK signaling and secretion of VCAM-1, enabling T-cell infiltration due to the breakdown of physical and chemical barriers built by CAFs in the TME [280]. The effect was markedly increased by combining FGFR blockade with checkpoint inhibitors in TNBCs [280]. Analogously, in a murine model with an activating mutation of FGFR2 in the mammary gland, FGF/FGFR2 signaling drove the development of TNBC, EMT, and tumor progression through STAT3 signaling and suppression of BRCA1 via the ERK-Yin Yang [YY]1 axis [281]. In this model, FGFR2 upregulated Programmed Death-Ligand (PD-L)1, and the combination of FGFR2 inhibition and immune checkpoint blockade killed cancer cells [281]. Despite these reports, BC metastases have relatively few infiltrating lymphocytes due to FGFR-mediated inhibition of the T-cell chemoattractant CXCL16, and consequently do not respond very well to immunotherapy. In the setting of a syngeneic BC mouse model, inhibition of FGFR signaling, either by covalent inhibition or by genetic depletion, had the effect of only delaying the growth of pulmonary metastases [282]. However, when combined with immune therapy, sequential FGFR inhibition followed by anti-PD-L1 therapy resulted in suppression of metastases [282].

It has also been documented that, as breast tumors grow and lay down stromal elements, compressive forces change the BC gene expression profile and the malignant phenotype [283]. However, low compressive forces in the TME also induce local immunosuppression via FGF-2 secretion from phenotypically modified tumor cells [279]. In primary patient-derived explants, low compression promotes a change in BC cells from a luminal to a mesenchymal, undifferentiated state [279]. In these explants that contain resident tumor-infiltrating lymphocytes, the altered tumor cells cause an upregulation of immunosuppressive cytokines IL-10 and TGFβ, and polarization of macrophages towards the tumor-supporting M2-type [279]. They also induce depletion of CD8+ effector memory T-cells, effects mediated by FGF-2 and prostaglandin E2 (PGE2) [279].

### 4.3. Inhibition of BC Progression by FGF Expression

#### 4.3.1. Implications of FGF Expression Inhibiting BC Progression

Cancer inhibitory effects were documented only from the expression of canonical FGF family members and were observed in multiple BC subtypes. Some results demonstrated effects in HR+ BC, some in TNBC, and some in multiple types. The anti-malignant effects were described in studies in multiple contexts. The following are the studies presented as categorized by tumor subtype.

#### 4.3.2. Multiple BC Molecular Subtypes or Subtypes Not Identified

Genetic variants in growth factors appear to influence BC risk through their combined effects in data analyzed from the Breast Cancer Health Disparities Study of 3592 cases and 4183 controls. The expression of the common genetic variant FGF-1 rs4912868 interacts with PDGF in being associated with improved survival after diagnosis in a variety of HR BC types of BC [37]. Recognizably, these associations involve genomic data obtained from bulk tumors containing a variety of cell types, identifying FGF family members as only some of the many other genes that have variants associated with BC risk and lack any biological data associated with their expression.

Transcripts of FGF-1, FGF-2, and FGF-7 have been detected in murine mammary preneoplasias, tumors, and immortal cell lines, but at levels less than those seen during normal mammary growth during development, not inconsistent with the report of FGF loss with progression towards invasive BC [65]. Indeed, the expression of FGF-2 is lost with malignant dedifferentiation [84]. FGF-2 and FGFR1 expression determined by immunofluorescence staining of archived pathologic samples from 55 random, anonymous patients with no patient or cancer identifiers, containing a total of 140 individual pathologic diagnoses, with many samples containing multiple stages of dedifferentiation on the same cut, demonstrated the expression of FGF-2 throughout benign and atypical dedifferentiation of mammary epithelial cells [84]. FGF-2 expression in epithelial cells was lost primarily in the step between carcinoma in situ and transformation to invasive cancer, with FGFR1 being expressed in all stages of dedifferentiation [84]. The strength of these investigations derives from the temporal associations in the changes in FGF expression and the histopathologic cellular attribution to FGF expression in tumors with different stages of dedifferentiation on the same pathologic section on a slide. The primary limitation is the fact, despite these strengths, that the data represent only associations.

Another study, whose strength lies in identifying expression in specific cell types, conducted gene expression profiling of epithelial cells manually microdissected from archival formalin-fixed paraffin-embedded blocks [284]. Samples from six patients with pure DCIS, six with synchronous lesions (IDC with an in situ component), six patients diagnosed with pure IDC, from areas with at least 70% of tumor cells having DCIS and IDC, and three non-neoplastic breast epithelium tissue areas were analyzed by gene expression profiling of 770 genes [284]. The molecular classification of the cells varied. The data demonstrated that three transcripts involved in cell invasion, FGF-2, Growth Arrest-Specific 1 (GAS1), a NOTCH receptor activator, and Secreted Frizzled-Related Protein 1 (SFRP1), a Wnt antagonist, were expressed in epithelial cells from the non-invasive group, but were downregulated in progression to later stages of invasive cancer [284]. These data confirm the loss of FGF-2 expression at the mRNA level and corroborate the immunohistochemical demonstration of the loss of immunoreactive FGF-2 with the transition from DCIS to invasive cancer [84]. Study limitations include the small number of samples, the expression of FGF-2 mRNA as one of among other genes regulated in the cell type, the lack of protein or molecular mechanism data and, as before, the association category of the information.

Messenger RNA was analyzed for the expression of FGF-1 and FGF-2 from whole frozen tissue samples from 22 patients with a variety of benign and hyperplastic tissue and 100 patients with carcinoma in situ and malignant BC of various histological types at surgery [82]. While the distribution of FGF expression in various cells in the tumors was not determined, and the variance was quite significant, the data convincingly demonstrated that the levels of both FGF-1 and FGF-2 mRNA were significantly higher in the benign group than in the malignant cancers [82]. The limitations of this study are its association design, the lack of cell type determination for the origin of the mRNA and the lack of protein expression, receptors or any molecular correlations.

Standing alongside studies where the data demonstrate a loss of FGF-2 with malignant progression are multiple publications demonstrating a contrary association of FGF-2 expression with a worse prognosis, exemplified by metastasis, DFS and OS. However, nuanced analysis is necessary to accept such conclusions. For example, in one gene expression profiling study using formalin-fixed paraffin-embedded (FFPE) TNBC tissues from a total of 36 patients with primary tumors and metastases from two cohorts, FGF family members, including FGF-7, were upregulated in primary tumors compared to matched metastatic lesions from among panels of genes differentially expressed [285]. In fact, in clinical analyses, tumors containing higher levels of FGF-2 determined by Northern and Western blots and immunohistochemistry from varied hormonal status tumors were associated with a less malignant phenotype in 51 patient samples [286], a lower S-phase in 1307 patients [36], a smaller tumor size, an earlier disease stage [36,287], and a better OS and DFS than patients with tumors containing lower levels of FGF-2 [36,286,287], although FGF-2 was not an independent prognostic indicator in a multivariate analysis [36]. Some of these studies have a number of strengths, including immunohistochemical discernment of cell type, protein expression, cell cycle effect, relatively large sample numbers and patient outcomes. Limitations include the correlative nature of the data, bulk tumor analysis in some studies, and lack of receptor data or molecular studies.

BC tumor samples from 66 patients with primary carcinomas with varied HR status and adjacent normal tissue and samples from benign tumors were analyzed by RT-PCR and Western blot [81]. Significantly less FGF-2 (*p* < 0.0001), FGFR1 and FGFR2 (*p* = 0.0078) were present in cancers than in normal tissue. Cancers appeared to have a significantly greater proportion of PCR product of the FGFR1 mRNA region between the third immunoglobulin-like domain and the tyrosine kinase domain, corresponding to the mesenchymally spliced c variant (*p* = 0.046), instead of the epithelially spliced b variant associated with proliferation and worse prognosis [81]. This study demonstrates a strong association between BC tumor tissue and lower FGF-2 and FGFR-1c and -2 compared with normal tissue, although immunohistochemistry was not done to identify the cell source. BC tumor samples from 51 patients were analyzed by RT-PCR, immunohistochemistry and Western blot, and patient outcomes were followed up for 84 months. FGF-2 was present in both malignant and non-malignant breast tissue, but expression was lower in malignant tissues using all three methods, which correlated well with each other. FGF-2 expression was observed by immunohistochemistry in non-malignant sample myoepithelial and epithelial cells but was generally lower or undetectable in malignant epithelial cells. While FGF-2 expression did not correlate with T stage, nodal status or ER status, patients with higher levels of FGF-2 expression had better overall DFS by Kaplan–Meier survival plots [286]. Immunohistochemical identification of epithelial sources and survival data provided strong correlative support for the anticancer effects of FGF-2 expression, but FGFR or molecular data were not obtained.

A study using an immunoassay to measure FGF-2 in tumor sample cytosols from 140 breast tumor samples found no significant differences in FGF-2 levels between malignant and non-malignant lesions [287]. However, in 124 cases of primary BC, there was an association of lower levels of FGF-2 (<400 pg/mg) with greater tumor size (*p* = 0.023) and progressive disease stage (*p* = 0.002). BC patients with low levels of FGF-2 had a significantly shorter DFS than patients with elevated FGF-2, with a median follow-up of 44.0 months (log-rank, *p* < 0.0001). Only FGF-2, T-stage and grade showed statistical significance in DFS by multivariate analysis [287]. The authors concluded that low FGF-2 levels are an independent prognostic indicator of poor prognosis and disease recurrence in BC [287]. These are strong association data on the anticancer effects of FGF-2 expression, but they are limited by the lack of cell source, FGF-2 protein size identification, FGFR and molecular studies.

None of these cited studies took into consideration treatment variables of patients in the study. In contrast, a study of 133 archived histologic samples of node-negative patients, pT1/2, N0/M0, and varied HR status, who did not receive hormone or cytotoxic systemic therapy because of the guidelines at the time for small, localized disease, measured FGF-2 by ELISA from whole samples to determine its impact on metastatic events [288]. There were 33 distant metastatic events in the population. Kaplan–Meier analysis of the prognostic significance of FGF-2 demonstrated that the occurrence of metastatic events was significantly lower in patients with tumor samples with high FGF-2 expression over a 130-month at-risk time period (*p* = 0.01) [288]. This study represents a compelling analysis of uniformly untreated patients with small, localized disease supporting the good prognostic impact of FGF-2 expression in BC, although it faces the same limitations as outlined above.

#### 4.3.3. HR+ BC Molecular Subtypes

As noted above, in a GWAS of patients of European ancestry for overall BC, FGF-20 showed a small genetically protective effect on overall BC and ER+ BC, although the analyses were from bulk tumors and the source of expression was not identified to be specifically from BC cells [14].

The investigations in the next two sections provide the strongest causal data that demonstrate anticancer effects and mechanisms of FGF-2 expression in both HR+ and HR− human BC cells in preclinical models. Studies in MCF-7 and T47D ER+ and MDA-MB-231 TNBC cells have demonstrated the impact of enforced expression on endowing a less malignant phenotype in vitro and in vivo in a murine xenograft model. Enforced expression of LMW and HMW FGF-2 in MCF-7 cells induced slower growth [18]. In this model, export of both LMW and HMW forms of FGF-2 competitively inhibited receptor binding by ^125^I-FGF-2. [18]. Expression of LMW and HMW FGF downregulated FGFR4 at the protein level but not at the mRNA level, without affecting FGFR1-3, while LMW FGF-2 expression alone had no impact on FGFR expression [18]. FGFR1 was constitutively phosphorylated in all FGF-2-expressing cells and able to be activated by exogenous FGF-2 in control cells. ERK was constitutively activated in the LMW cells but was not activated, nor able to be activated by exogenous FGF-2 in HMW cells. p27^Kip1^ was constitutively upregulated in both cell types expressing FGF moieties compared with controls; p21^Waf1^ was only upregulated in the LMW cells; and Rb was dephosphorylated only in the LMW cells, as well. The mechanism of cell cycle inhibition was mediated through different mechanisms in the cells expressing the different isoforms [18]. Enforced expression of LMW FGF-2 induces a more differentiated phenotype in T47D BC, another ER+ cell line, as indicated by decreased proliferation, migration and invasion, induction of branching morphogenesis in Matrigel and impaired anchorage-independent colony formation [106]. Cells expressing both HMW and LMW forms of FGF-2 together lost the ability to have their motility stimulated by either exogenous FGF-2 or their invasion stimulated by added estrogen [106]. Enforced expression of LMW and HMW FGF-2 in MCF-7 cells also decreased the expression of Bcl-2 and increased the rate of apoptosis in response to chemotherapy agents Etoposide and 5-fluorouracil in vitro [289].

In another study of retrovirally transduced enforced expression of FGF-2 in MCF-7 cells, the transduced FGF-2 was expressed in the cytoplasm and nuclei, and was also released into the culture medium [290]. Transduced FGF-2 induced branching in both plastic culture dishes and Matrigel. Blocking antibodies to FGF-2, integrins α2, α3 and β1 abolished branching in Matrigel and anchorage-independent growth in soft agar. Cells expressing FGF-2 had a 1.6- to 3.2-fold reduction in tumor volume compared to parental controls, and reduced estrogen receptor-α (ERα) expression both in vitro and in vivo. The authors concluded that FGF-2 expression in MCF-7 cells induced a more benign phenotype in vitro and in vivo [290]. The data also demonstrated that the induction of branching morphogenesis is dependent on extracellular FGF-2 signaling in addition to intracellular expression. Physical interactions of integrins α2, α5 and β1 with FGFR2 are key to integrin-driven adhesion and migration in normal mammary epithelial cells, and are disrupted by carcinogenic progression and loss of FGFR2 and concomitant integrin expression [291]. Receptor activation by environmental FGF-2 restores expression of integrins lost with dedifferentiation in ER+ BC cells [39]. FGF-2 induces branching morphogenesis in mammary epithelial cells through FGFR1-mediated signaling and activation of ERK1/2 [292]. FGFR1-induced branching and activation of ERK1/2 depend on its binding of DC-SCRIPT (transcription regulator Zfp366), the expression of which is lost in malignant transformation of ER+ BC, along with the capacity of FGF-2 to activate ERK1/2 [292].

#### 4.3.4. TNBCs

The enforced expression of LMW FGF-2 in MDA-MB-231 TNBC cells also inhibits motility, an effect mediated by circumferential activation of focal adhesion kinases and cortical rearrangement of F-actin stress fibers [107]. MDA-MB-231 cells expressing LMW and HMW FGF-2 did not export any of the isoforms and maintained unchanged expression of FGFR1 and FGFR3. The doubling time of cells expressing the two FGF-2 isoforms was not different from that of controls in tissue culture. However, cells expressing both isoforms had decreased anchorage-independent growth in soft agar, but only cell constructs expressing the LMW FGF-2 formed fewer and smaller tumors in athymic mice [105]. Tumor cells acquired the ability to export FGF-2. Also, an apparent selective pressure on the inhibitory effects of FGF-2 expression on tumors resulted in some larger tumors that lost their expression of transduced FGF-2 [105]. Both classes of cell constructs had decreased motility, but only the LMW constructs had decreased invasion, which was attributed to diminished motility [107]. The combined data from these studies demonstrate that enforced expression of FGF-2 induces a more differentiated phenotype in both ER+ and TNBC cells. FGF-2 also represses the expression of periostin, a matricellular protein, which is detected in about half of HER2+ breast tumors and is correlated with more aggressive behavior, through a PKC-dependent pathway involving crosstalk among FGFR, TGFβ and the PI3K/AKT pathway [293]. Periostin is involved in angiogenesis, invasion, survival and metastasis [293]. The limitations of all of these preclinical investigations are the recognition that data in preclinical systems does not faithfully recapitulate the behavior of cancers in patients.

In the sections above, all the studies provide data supporting the inhibitory effects of canonical FGF family member expression in BC, but references [18,84,105,106,107,286,287,288,290] provide the most compelling evidence, in consideration of their varied limitations, for the hypothesis presented by this review that expression of FGF by BC cells promotes a less malignant phenotype.

#### 4.3.5. Key Takeaways from the Data on the Cancer-Inhibiting Effects of FGF Expression

Data in this section support associations and causative effects between FGF expression and less malignant phenotypes in BC. The following conclusions summarize compelling evidence and circumstances that confer these effects.

1. Expression of only the canonical FGF category members FGF-1, -2, -7, and -20 is associated with a less malignant phenotype. Enforced expression of only FGF-2 was reported to induce a less malignant phenotype.
2. Co-expression of a common FGF-1 genetic variant and PDGF is associated with a better prognosis.
3. Cell-based and cell-specific tissue-based analyses of human samples demonstrated an association between progressive loss of FGF-1 and -2 and mammary epithelial cell dedifferentiation. The greatest loss was observed at the step between DCIS and invasive cancer. Associated losses of structural duct-enveloping Laminin 5, that of a NOTCH receptor activator, and a Wnt antagonist were observed.
4. FGFR1 was observed in both pre-malignant and malignant epithelial tissue during dedifferentiation. FGFR1 was higher in tumor cells than in adjacent tissue and more likely mesenchymally spliced. FGFR amplifications, mutations, splicing or levels of expression were not addressed in most studies.
5. Greater FGF expression was associated with favorable tumor characteristics.
  - FGF-2 was associated with earlier stage, smaller size, no metastases, better OS and DFS in multiple studies and multiple BC subtypes. Lower FGF-2 expression levels were consistently associated with larger tumors, later stage, poor DFS and prognosis.
  - FGF-7 expression was higher in primary tumors than paired metastases.
  - FGF-20 showed a small genetic protective effect in overall BC and ER+ BC.
  - A seminal study addressing untreated early-stage patients demonstrated an association with significantly lower recurrence with metastatic events in patients with high FGF-2 expressing tumors.
6. Enforced expression of LMW and HMW FGF-2 in ER+ cells and in TNBC cells induced a less malignant phenotype, differentiation in vitro and in murine xenografts. FGF expression inhibited proliferation through CDK inhibitors, induced differentiation, downregulated Bcl-2 and endowed a greater sensitivity to chemotherapy in ER+ cells.
7. Enforced expression of combined LMW and HMW FGF-2 downregulated FGFR4 expression, which was shown before to promote cancer progression. Enforced LMW expression had no effect on receptors.
8. Export of HMW FGF-2 competitively inhibits LMW FGF-2 FGFR binding and its ability to activate ERK 2 in cells expressing HWM FGF-2. Thus, HWM FGF induced a two-fold mechanism of FGFR signaling inhibition and intracellular cell cycle inhibition.
9. Enforced expression of FGF-2 induces integrin expression that contributes to cell differentiation and a less malignant phenotype in vitro and in vivo in multiple cell types.
10. Key limitations: The sum of these studies is, to date, the most complete demonstration of some of the mechanisms of combined intracellular expression signaling, export and receptor-mediated signaling in coordination with integrins and other pathways. Each study demonstrates only some of the elements of the dual mechanisms outlined in the classical FGF literature in the first part of this manuscript.
  - Some compelling studies demonstrating an association between loss of FGF expression and malignant progression were conducted by measuring FGF expression in epithelial cells or epithelial-rich segments of patient tissue, not individual cells.
  - Studies that measured bulk malignant and benign tissue had greater variance, but also demonstrated this effect upon careful analysis.
  - Determination of prognosis was based on population-averaged data and did not consider other variables, including treatment. The one study that addressed FGF-2 expression in untreated patients was a convincing isolation of this variable and a convincing demonstration of the association of FGF-2 expression with a cancer inhibitory effect in a clinical setting.
  - Bulk tumor studies did not consider the microenvironment, location of the sample in the tumor, prior therapy, co-signaling by other receptors or the structural microenvironment, as in previous discussions of the approach. However, survival studies in specific, defined populations were able to systematically correlate the upper half and lower half of the population survival curves with statistically significant outcomes correlating with FGF expression, albeit with large variance.

Overall, the enforced expression causative preclinical studies were most illuminating and convincing. They demonstrated inhibitory, differentiation, and survival signaling and phenotype in vitro and in vivo, in both ER+ and TNBCs. They identified informative biology regarding FGFR competitive inhibition by exported HMW FGF-2 and induction of cyclin-dependent kinase inhibitors. This line of investigation needs follow-up to further clarify the mechanisms. Knockout and knock-in experiments with various FGFR subtypes and splice isoforms will clarify the mechanisms of inhibition and differentiation. Co-signaling by integrins and FGF-2 or classical integrin ligands will elucidate the role of FGF in the microenvironment. These preclinical studies will permit clinical trial design for FGFR signal inhibition to increase the chances for success. Table 4 outlines the experimental models, the components of the dual signaling hypothesis partially supported by the studies cited, and key limitations of the studies.

## 5. Conclusions

A total of 17 of the 23 members of the FGF family from three general categories play roles in normal and malignant breast physiology. FGF proteins function by binding and signaling through FGF receptors via classical signal transduction and internalization followed by intracellular signaling. They also signal directly after cytoplasmic synthesis and subcellular localization. Combined receptor-mediated and intracellular signaling are required for cancer promotion.

**Figure 3:**
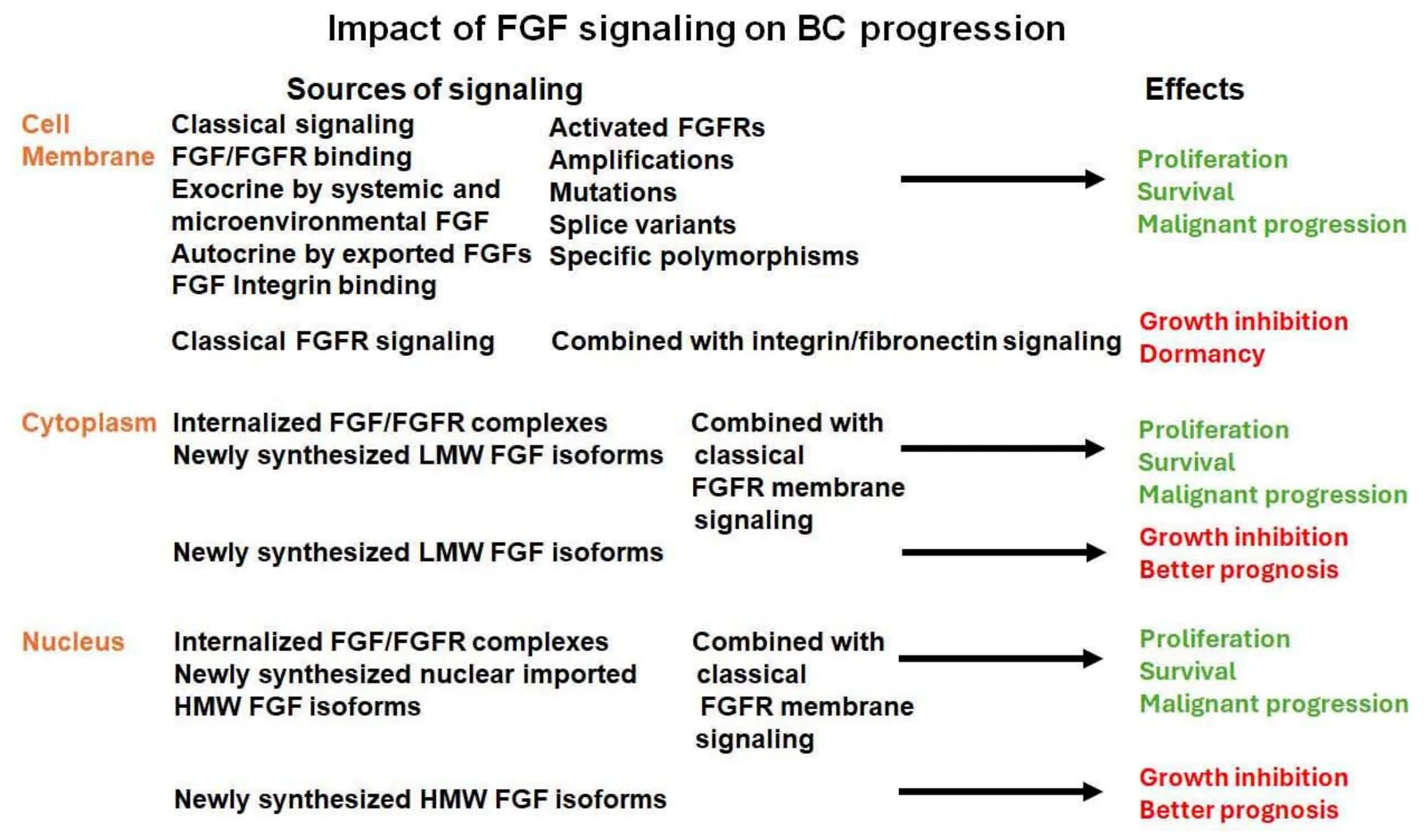
Impact of FGF signaling in BC progression.

Expression of FGF family members by breast cancer cells has been reported to induce pro-cancerous effects as well as cancer inhibitory effects. The exceptionally large volume of literature addressing the effects of FGF family members in breast cancer presents an endless variety of experimental variables that impact the observed effects on BC. Many of these are outlined throughout this document in take-away segments at the ends of each major section and are summarized in Figure 3.

Clinical and preclinical association studies are directly relevant to cancer behavior, but they are only correlative. Experimental design differences in studies involving patients, tumor samples, experimental design and measurement of FGF expression have generated a great deal of variability. Systematic and specific measurements demonstrating loss of FGF expression with malignant progression have been supported by preclinical causative data from enforced expression or knockout studies demonstrating the mechanisms of these effects. On the other hand, the massive data associating FGF family member expression with worse cancer behavior were confounded by a significant number of variables, including dual signaling by exogenous FGF and other proteins from the cancer microenvironment, affecting their impact on cancer behavior. These effects are consistent internally within experimental design, but the context of the experimental circumstance, the microenvironment, and what is being measured are diverse and are often lacking in the control of confounding variables. These elements must be considered in designing future experiments.

The combination of clinical associations with causative preclinical data will permit the design of interventional clinical trials to identify the optimal approaches to blocking FGFR signaling to inhibit BC progression. Such trials have been disappointing to date in BC because of likely inappropriate patient selection for the tumor characteristics and corresponding appropriate signaling to inhibit. The claim that the association of growth factor signaling with BC risk is modest may not necessarily be true in all circumstances. Defining the appropriate scenarios in preclinical investigations will provide helpful data for clinical trial design.

## Figures and Tables

**Table 1:** FGF family members and categories and reported roles in the mammary gland.

Category	Canonical					Endocrine	Intracellular
Subfamily	1	4	7	8	9	15/19	11
FGFs	FGF-1, -2	FGF-4, -5, -6	FGF-3, -7, -10, -22	FGF-8, -17, -18	FGF-9, -16, -20	FGF-15/19, -21, -23	FGF-11, -12, -13, -14
FGFs with reported BC effects	FGF-1, -2	FGF-4, -5	FGF- 3, -7, -10, -22	FGF-8, -17, -18	FGF-9	FGF-19, -21, -23	FGF-11, -13

**Table 2:** Sources of heterogeneity in FGFR signaling.

Signaling Component	Source of Variability
FGF family member	Category, subfamily, member, translated isoform
FGF source	FGF species available for receptor binding from microenvironment or from autocrine export
Cofactors	Heparan sulfate proteoglycans, Klotho from the microenvironment
FGFRs expressed by cell	FGFR1-4, splice variants, receptor mutations, amplifications, polymorphisms
Integrins expressed by cell	Paired integrins expressed and available for binding of FGF isoforms and of structural proteins, availability of specific structural proteins from the microenvironment
Other membrane and nuclear receptors	Crosstalk between FGFR and other signaling and availability of other receptor ligands
Cell type	FGF signaling in the context of the active programs defining the cell type.
Import of FGF/FGFR membrane complexes to the cytoplasm and/or nucleus	Phosphorylation of complexes, direct interaction with signaling molecules in the cytoplasm, nuclear import, direct nuclear signaling, and re-export

**Table 3:** Critical analysis of data supporting pro-tumorigenic effects of FGF expression.

Experimental Model	Mode of Support	Model Component Supported	Limitation	Refs.
Databases w/o or w/correlative cell line data	Associations: - FGF-1, -2, -4, -7, -20, -23 expression, FGFR2 internalization & malignancy	- High FGF proteins or mRNA, low FGFR2 and poor prognosis	- Large databases - No data on FGF cellular location, export, receptor variant, or causation	[14,232,248]
	- Proof of autocrine pro-malignant FGFR1 signaling in correlative cell line	- Dual signaling; FGF-2 expression + autocrine FGFR1 canonical sign.	- Preclinical-only proof of dual signaling	
Patient tumor samples w/o cell line support data	Associations: - FGF-3, -4, -5, -18, -19 expression & worse clinical, molecular characteristics - FGF-11, FGFR1 and 4 expression in tumor cells	- Elevated FGF mRNA & poor prognosis and pro-malignant effects - FGF, FGFR protein expression a/w dual signaling in malignancy.	- One gene among many - Bulk tumor tissue, cell source of FGFs unknown - mRNA expression only, no protein or FGFR data - No data on phenotype	[7,13,78,170,171]
Patient tumor samples w/cell line correlative data	Associations: - Higher FGF-4, -5, -13, -16 RNA in bulk tumor than in adjacent tissue; a/w worse disease, worse survival - Same expr. in primary tumor & and normal mammary tissue suggest paracrine effects. - High FGF-10 only in tumors of high epithelial/stromal ratios; stromal FGF source in mamm. dev. - FGFR2b in epithelial cells: supports dual signaling req. for pro-malignant effects - IHC FGF-1 in epith. cells, fibroblasts, not benign cells; FGFR3b in epith. cell nuclei - Primary tumor FGF-19 higher after paclitaxel Rx in patients who developed metastases, a/w lower RFS	- High epithelial FGF, FGFR, nuclear translocation, autocrine & stromal FGF sources; support dual signaling - FGF expression a/w resistance, metastases, poor RFS	- RNA measured only, cellular source not determined; no protein or FGFR data. - Associations of FGF and FGFRs with malignancy, but no receptor or internal signaling, localization, FGFR internalization, or other coordinated signaling or cancer variables.	[8,12,169,229,233,240,241,251]
	Causative effects: - Cell line enforced expression/K/O FGF-4, -5, -13; causative data for metastasis, not local growth - High FGF-1, FGFR in epithelial cells & paracrine stromal FGF source cause malignant behavior - Enforced expression of FGF-16 induced more malignant phenotype in vitro and in vivo, EMT and metabolic reprogramming - Cell line data confirmed association of FGF-1 and autocrine FGFR3b signaling - Strong causative role for FGF-19 in malignancy in vitro and in vivo, a/w pFGFR4, stress response - K/O of FGF-19: proof of FGF role in malignant transformation in vitro and in vivo via FGFR4/Akt	- FGF expression causes metastasis, metabolic reprogramming, EMT - Stress response signaling -Autocrine activation by exported FGF. - K/Os provide proof for dual intracellular & canonical FGFR4 signaling necessary	- No data on subcellular localization, export, receptors, autocrine or paracrine signaling, downstream effectors; some not mechanistic; - Preclinical; do not recapitulate the behavior of human BCs in patients - Small studies - No FGFR inhibition or subcellular localization studies - No other pathways investigated	
Cell lines	Associations: - FGF-1, -19, -21, -23 expression in bulk mouse bone homing tumors	FGF mRNA in bulk bone homing TNBC xenograft tumors in a panel of gene arrays	- Association - Many other genes regulated - No mechanisms noted	[11,14,15]
	Causative effects: - Enforced FGF-18 expression/inhibition induces/inhibits signaling, malignancy in vitro/in vivo - Enforced expression of FGF-1, -4 induces metastatic disease, angiogenesis and oxygenated tumors - Autocrine FGF-2/FGFR signaling drives in vivo, in vitro 3D but not 2D growth - CAFs induce cell invasion through FGF-7, -9 signaling - In vitro FGF-5 positive feedback loop between stroma and epithelial cells	- High FGF protein in BC cell lines induces malignancy - Combined cellular FGF and paracrine TME FGF/FGFR canonical signaling cause malignant effects	- Lacking receptor features, FGF export, autocrine signaling, FGFR activation, internalization, intracellular signaling are not addressed - Preclinical data does not reproduce human tumors	[80,236,237,238,239,246,247,249,250,252]
Mouse mammary virus tumor model	Association: FGF-3, -10 expr. & autocrine FGFR1 STAT3 and Akt signaling induce mammary tumors	- Dual roles of intrinsic expression and autocrine FGFR1 STAT3 & Akt signaling	- Association - Mouse MMTV model not representative of human BC	[69,241]

Blue font indicates causal evidence.

**Table 4:** Critical analysis of data supporting antitumor effects of FGF expression.

Experimental Model	Mode of Support	Model Component Supported	Limitations	Refs.
Databases w/o correlative cell line data	Associations: - FGF-1 common genetic variant interacting with PDGF a/w better survival - FGF-20 a/w a small genetically protective effect from BC in a GWAS	- FGF expression a/w other signaling pathways and patient survival - FGF expression a/w BC protective effect	- Associations only - One of many FGFs in GWAS panel a/w less risk - No data on protein, FGFRs, signaling, paracrine stimulation - Bulk tumor analysis, cell source not determined - No mechanistic insight	[14,37]
Tumor samples w/o cell line support data	Associations: - Lower FGF-1, -2, -7 mRNA in mouse tumors than in mamm. dev. - Lower FGF-2 mRNA among many genes in panel in BC progr. patients - Lower FGF-1, -2 mRNA in patient CIS and IBC - Lower FGF-2, FGFR1 & 2 mRNA & protein in bulk tumors than benign tissue; c/w expr. loss w/malign. - Higher FGFR1c/FGFR1b ratio a/w worse prognosis - Higher FGF-7 mRNA in primary tumors than mets. - High early tumor FGF-2 protein in untreated pats. a/w fewer metastatic events -FGF-2 mRNA & protein in bulk tumors a/w better tumor biol. & pat. outcomes	- Loss of FGF-2 expression with mammary epithelium progressive dedifferentiation to BC - Lower FGF in metastases than tumor - Higher FGF expression and better outcomes - Low FGF-2 is an independ. indicator of poor prog. & recur. - Convincing association of FGF expr. & less malig. behavior: - Multimodal experimental designs - Temporal FGF expr. loss a/w stepwise prog. in primary tum. & mets - Statistical validation	- Associations only between FGF loss and malignant transform., no interventional studies - mRNA only, no protein data in some studies - Cellular source of RNA not determined, but protein expr. localized by IHC in tissue samples - Association of FGF expression and better patient outcomes - No mechanisms or molecular context on cancer inhib. signaling - Most studies did not consider treatment differences	[36,65,81,82,84,284,285,286,287,288,293]
Cell lines	Causative effects: - Inhibitory effect of enforced LMW & HMW FGF-2 expr. in cell lines; less malignant behavior in vitro and in vivo - LMW, HMW FGF-2 export in HR+ human BC cells - HMW & LMW competitively inhibit FGFR- and ER-mediated effects - Enforced expression and export of HMW FGF-2 downregulates FGFR4, endows less malignant phenotypes in vitro and in vivo in mouse model	- LMW & HMW FGF-2 expr. inhibits BC cells - Export of HMW and LMW FGF-2 - Competitive inhib. of FGFR activation - HWM FGF-2 expr. inhibits FGFR-med. FGF-2- or E2-induced motility/invasion - HMW FGF-2 expr. downregulates FGFR4 - FGF-2 expr. endows less malig. phenotypes in vitro & xenografts -HMW FGF-2 nuclear expr. induces p27^Kip1^ & cell cycle inhibition - LMW FGF-2 expr. induces p21^Waf1^ & cell cycle inhibition - HMW FGF-2 induces treatment resistance by downregulating Bcl-2 - LMW & HMW FGF-2 combined w/ integrins induce branching morphogenesis - FGF-2/FGFR1 ERK & Zfp366 signaling restores integrins lost with malignant progr. - FGF-2 expression decreases xenograft volume and ERα expr. - Expression of FGF-2 in HR+ BC induces a more benign phenotype - Evidence for required FGF expression + FGFR-mediated FGF-2 + integrin signaling for tumor suppression	- Each study demonstr. only subset of elements of dual mechanisms for cancer promotion - Limited number of cell lines must be expanded for all molecular types - Preclinical studies only - FGFR and FGF & trafficking, dual signal. mechanisms, crosstalk with microenvironment and other pathways not characterized - Absence of human BC interventional trials with specific inclusion criteria, molecular components investigating expression, localization, export, receptor characteristics, internalization, internalization, intracellular signaling crosstalk with adhesion molecules and other signal pathways, inhibition of pro-cancer signaling and patient survival advantage	[18,39,106,289,290,291,292]

Blue font indicates causal evidence.

## Data Availability

No new data were created or analyzed in this study. Data sharing is not applicable to this article.
